# Assessment of Genetic Diversity, Population Structure, and Evolutionary Relationship of Uncharacterized Genes in a Novel Germplasm Collection of Diploid and Allotetraploid *Gossypium* Accessions Using EST and Genomic SSR Markers

**DOI:** 10.3390/ijms19082401

**Published:** 2018-08-14

**Authors:** Allah Ditta, Zhongli Zhou, Xiaoyan Cai, Xingxing Wang, Kiflom Weldu Okubazghi, Muhammad Shehzad, Yanchao Xu, Yuqing Hou, Muhammad Sajid Iqbal, Muhammad Kashif Riaz Khan, Kunbo Wang, Fang Liu

**Affiliations:** 1 State Key Laboratory of Cotton Biology/Institute of Cotton Research, Chinese Academy of Agricultural Sciences, Anyang 455000, Henan, China; adbotanist@yahoo.com (A.D.); zhonglizhou@163.com (Z.Z.); cxycri@163.com (X.C.); wxx1991@126.com (X.W.); wediweldu81@yahoo.com (K.W.O.); mshehzad534@gmail.com (M.S.); xuyanchao2016@163.com (Y.X.); houyuqing18@163.com (Y.H.); sajidses@hotmail.com (M.S.I.); 2 Nuclear Institute for Agriculture and Biology (NIAB), Jhang Road, Faisalabad 38000, Punjab, Pakistan; mkrkhan@gmail.com; 3 Hamelmalo Agricultural College, P.O. Box 397, Keren, Eritrea

**Keywords:** novel accessions, PIC, PCR, EST-gSSRs, genes, genetic distance

## Abstract

This study evaluated the genetic diversity and population structures in a novel cotton germplasm collection comprising 132 diploids, including *Glossypium klotzschianum* and allotetraploid cotton accessions, including *Glossypium barbadense*, *Glossypium darwinii*, *Glossypium tomentosum*, *Glossypium ekmanianum*, and *Glossypium stephensii*, from Santa Cruz, Isabella, San Cristobal, Hawaiian, Dominican Republic, and Wake Atoll islands. A total of 111 expressed sequence tag (EST) and genomic simple sequence repeat (gSSR) markers produced 382 polymorphic loci with an average of 3.44 polymorphic alleles per SSR marker. Polymorphism information content values counted 0.08 to 0.82 with an average of 0.56. Analysis of a genetic distance matrix revealed values of 0.003 to 0.53 with an average of 0.33 in the wild cotton collection. Phylogenetic analysis supported the subgroups identified by STRUCTURE and corresponds well with the results of principal coordinate analysis with a cumulative variation of 45.65%. A total of 123 unique alleles were observed among all accessions and 31 identified only in *G. ekmanianum*. Analysis of molecular variance revealed highly significant variation between the six groups identified by structure analysis with 49% of the total variation and 51% of the variation was due to diversity within the groups. The highest genetic differentiation among tetraploid populations was observed between accessions from the Hawaiian and Santa Cruz regions with a pairwise F_ST_ of 0.752 (*p* < 0.001). DUF819 containing an uncharacterized gene named yjcL linked to genomic markers has been found to be highly related to tryptophan-aspartic acid (W-D) repeats in a superfamily of genes. The RNA sequence expression data of the yjcL-linked gene Gh_A09G2500 was found to be upregulated under drought and salt stress conditions. The existence of genetic diversity, characterization of genes and variation in novel germplasm collection will be a landmark addition to the genetic study of cotton germplasm.

## 1. Introduction

The leading natural fiber in the world is a product of cotton crops. Cotton is placed in the taxonomic order with the genus *Gossypium* and has broad phenotypic diversity, which includes more than 50 species [1,2,3]. There are now 7 tetraploid and 46 diploid cotton species after molecular confirmation and taxonomic designated two new tetraploid ones, i.e., *Gossypium ekmanianum* (AD6) and *Gossypium stephensii* (AD7) [3,4,5,6]. Among those, four are cultivated throughout the world: two of these species are diploids (2*n* = 2*x* = 26) and two are allotetraploids (2*n* = 4*x* = 52). Global cotton production is manifested from the two allotetraploid species *Gossypium hirsutum* and *Gossypium barbadense* [7,8,9].

Data on allotetraploid cotton evolution indicates that the seven tetraploid cottons evolved about 1.5 million years ago by hybridization of the Old world cotton *Gossypium herbaceum* (A_1_ genome) and the New world cotton *Gossypium raimondii* (D_5_ genome) as a consequence of subsequent diploidization and domestication [3,4,8,10,11,12]. G*ossypium hirsutum*, also called “Upland cotton”, represents 90% of global cotton fiber production [13], while *Gossypium barbadense* (also known as Pima) is valued for its extra-long staple fiber source, is domesticated in North-West South America, has its native origin in Egypt, and contributes around 8% of total world lint [9]. Wild *Gossypium darwinii* originated from Galapagos Island and, relative to *G. barbadense,* also has good fiber fineness characteristics and is a rich source of resistance to *fusarium* and *verticillum* wilts [14]. The d-genome *Gossypium klotzschianum*, having glabrous seed coverings, evolved through long-distance dispersals, is endemic to Galapagos Island, and is considered a New-World d-genome diploid along with *G. raimondii*. *Gossypium tomentosum* is drought-tolerant, native to a Hawaiian Island, and has a more diffuse population structure falling typically as scattered individuals and small populations on several islands. *G. tomentosum* (AD3), *G. darwinii* (AD5), *G. ekmanianum* (AD6), and *G. stephensii* (AD7) are wild and are not grown commercially [1,2,15,16]. Wendel and Percy analyzed 58 *G. darwinii* accessions from six islands using 17 isozyme markers and identified a high genetic diversity level within these accessions and relationships with *G. barbadense* and *G. hirsutum* genomes. This classic study suggested that *G. darwinii* and *G. barbadense* are separated and each has a distinct genome [17].

The genetic diversity of different plant species is an essential element for crop production in agriculture, including cotton. Genetic variation in the *Gossypium* species is widespread, covering large geographic and ecological niches. It is a vital source of conserved genetic diversity in situ in Mexico for cotton origin [18,19] and is preserved ex situ within worldwide cotton germplasm collections and materials of breeding programs. The productivity of cotton and future efforts to improve cotton depend to a large extent on the elucidation of genetic diversity in cotton genetic stocks and their effective utilization in cotton improvement programs [20].

The narrow genetic background of Upland cotton has become a major concern as low genetic diversity gives rise to stagnant yield and quality of breeding. The elite breeding programs cannot make robust inferences without using the unexploited standing genetic variation of archaic cultivars typically associated with wild accessions [14,21,22]. The characterization of genetic diversity between and within groups enables us to find heterozygous groups, understand population structures, and isolate a core set of lines for genetic analysis studies in cotton. A multitude of studies indicate the extensive usage of model-based structure analysis for investigating genetic diversity in cotton [22,23]. Genetic diversity estimates have been established using genotypic data and DNA-based molecular markers [24,25,26,27,28]. Molecular markers are more reliable since they can directly determine allelic diversity and give robust estimates of genetic distances.

The DNA-based markers used for determining genetic diversity in cotton include restriction fragment length polymorphisms (RFLPs) [29], random amplified polymorphic DNA (RAPD) [30,31,32], amplified fragment length polymorphisms (AFLPs) [33], simple sequence repeat (SSR) [9,34,35,36], expressed sequence tags (ESTs) [37], inter-simple sequence repeat (ISSR) [38,39], and single nucleotide polymorphisms (SNPs) [40]. Compared with other biomarkers, SSR has advantages that include more reproducibility, co-dominant inheritance, distribution throughout the genome, and its being highly transferable, informative, and reliable [41].

Although data from several studies implicates the marker-based estimation of genetic diversity in cotton, the majority of those remain bound to the number of accessions included or the number of markers used to describe genetic diversity [42]. Recently, an effort has been made by Kirungu et al. [43] to explore the important genes linked to SSR markers by constructing a genetic linkage map between *Gossypium davidsonii* and *G. klotzschianum*. Similarly, a study of gene diversity, their functionality, and especially the diagnosis of uncharacterized domains of proteins in developing the evolutionary relationship among cotton accessions will be fruitful for exploring the mystery of cotton evolution. Among all protein domains with a unique structure and functions, nearly more than 20% are currently described as “domains of unknown function” (DUFs). They are often overlooked as irrelevant as many of them are found in only a few genomes. Approximately 2700 DUFs exist in bacteria as compared to eukaryotes, which have only 1500. More than 800 DUFs have been found to be common in bacteria and eukaryotes, and about 300 of these are also present in archaea. Evolutionary conservation suggests that many of these DUFs are important in biology as they mostly represent single-domain proteins, clearly establishing the biological importance of DUFs [44].

The importance of prioritizing DUFs has been recognized in various experimental and/or computational characterization efforts [45,46,47,48]. We identified DUF819 (PF005684), which is not only highly conserved but also plays an important role against biotic and abiotic stress, among four sequenced cotton species by using the WDR (PF00400) superfamily as reference-genome-sequenced proteins. Genome-wide characterization of WD-repeats, also known as tryptophan-aspartic acid or the W-D superfamily, has only been conducted in Arabidopsis and Cucumber [49,50] till now. Therefore, a comprehensive study comprising a wide collection of germplasms, more efficient genotyping, and collective genomic platforms is required to measure the overall genetic diversity in diploid and allotetraploid cotton, which will help overcome the future challenges of the gene pool’s disastrous escape.

The objectives of this study were to explore the genetic diversity and evolutionary relationship among the domains of uncharacterized proteins in natural diploid and allotetraploid cotton germplasm resources and to analyze the population structures to maximize estimations about the accessions of cotton present in a wild nursery of China for their efficient utilization in cotton-breeding programs.

## 2. Results

### 2.1. SSR Marker Analysis

Among a total of 853 SSR primer pairs used for genotyping 132 accessions, 205 primer pairs were found to be polymorphic with a polymorphism rate of 24%. Accessions with more than 5% missing data were removed and 94 SSRs were dropped; the selected 111 SSR primer pairs can be scored confidently and read clearly on PCR products. Data for monomorphic loci were also excluded from the analysis. Data generated from the selected 111 SSR primer pairs was analyzed. Among 132 accessions, a total of 382 SSR alleles were detected as marker loci with an average of 3.44 alleles per SSR ranging from 2 to 8. All 382 SSR loci were found to be polymorphic. The average polymorphism information content (PIC) value for SSRs was 0.555 with a range of 0.078 to 0.821, and the major allele frequency was 0.738 ranging from 0.541 to 0.959 for the complete panel. Seventy-six (68.468%) SSR markers in total were found to be highly informative with a PIC value ≥0.50, 29 (26.126%) were moderately informative with PICs value ≥0.25 and <0.50, and 6 (5.405%) were least informative with a PIC value <0.25. A summary of marker statistics for *G. hirsutum* accessions is listed in Supplementary Table S2.

### 2.2. Unique Alleles

Among the 382 alleles detected in the studied accessions, 123 alleles were found and were termed as unique alleles (Supplementary Table S3). A high percentage (17.51%) of unique alleles was observed in *G. ekmanianum* genotypes (Table 1). Twenty-five unique alleles were found in two accessions of *G. hirsutum*. Nineteen, 13, 18, 13, and 4 unique alleles were observed in *G. barbadense*, *G. tomentosum*, *G. darwinii*, *G. klotzschianum,* and *G. stephensii*, respectively. *G. ekmanianum* had the highest number (31) of unique alleles, which were collected from the Dominican Republic, National Plant Germplasm System (NPGS) USA (Supplementary Table S3). These unique alleles are an important genetic resource for cotton and have never been studied before.

### 2.3. Common Alleles

Common alleles were estimated to understand the phenomenon of cotton evolution and the gene flow mechanism. All six species of cotton considered in this study have common alleles at 114 loci, keeping *G. hirsutum* as fixed. The SSR marker DPL0330-A showed the maximum number of common alleles (124) among all tetraploid cottons except for *G. klotzschianum*, which is diploid, while DPL0249-C showed the minimum number of common alleles, which were only found in *G. barbadense*, *G. klotzschianum*, and *G. hirsutum*. The number of common alleles ranged from 21 to 123 in all six species of *Gossypium*. In this investigation, a total of 459 common loci were observed. Eighty-eight, 101, 82, 91, 67, and 30 loci having common alleles specific to *G. hirsutum* were observed in *G. barbadense*, *G. darwinii*, *G. tomentosum*, *G. ekmanianum*, *G. stephensii,* and *G. klotzschianum,* respectively (Supplementary Table S4). These *G. hirsutum*-specific alleles were amplified by 85 out of 111 SSR markers. The presence of *G. hirsutum*-specific alleles in all six species of *Gossypium* indicated a high level of natural introgression. The level of introgression was found to vary among these wild-type accessions [36,51].

### 2.4. Analysis of Population Structures

**Figure 1:**
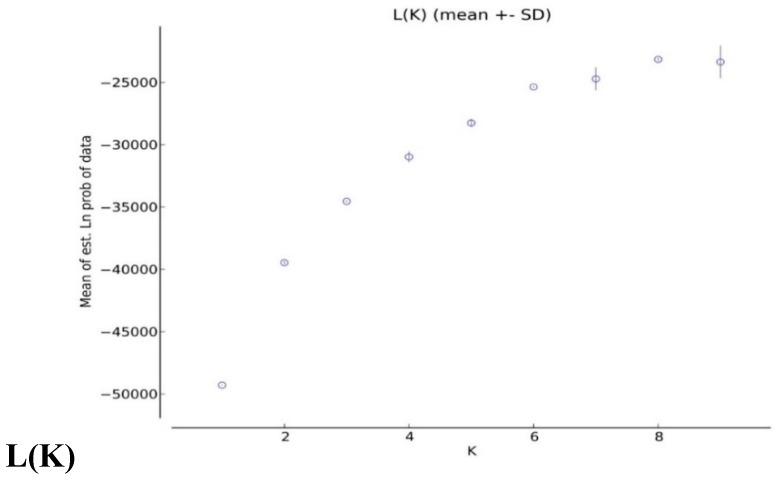
K means for 132 accessions.

**Figure 2:**
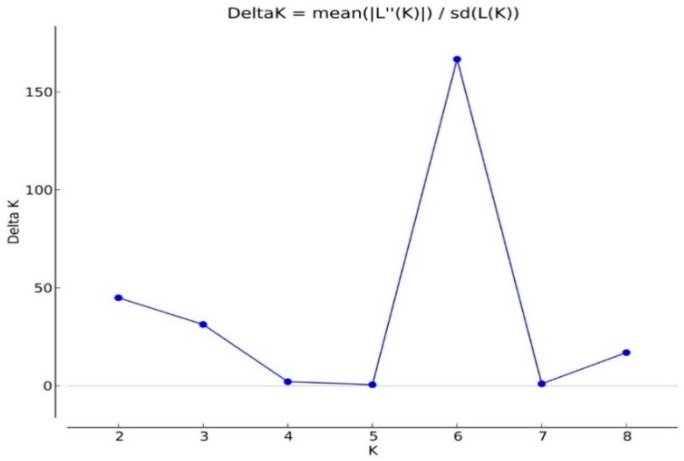
Delta K for 132 accessions.

**Figure 3:**
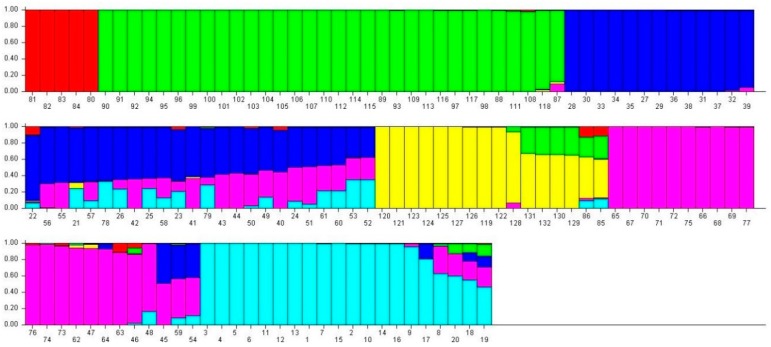
Q plot showing clustering of 132 accessions in 6 subpopulations based on an analysis of genotypic data using STRUCTURE software ver. 2.2. Each accession is indicated by vertical bars. The color subsections within each vertical bar represent the membership coefficient (Q) of the accession to different colors. Six groups were identified. The identified groups are I (red), II (lime), III (Blue), IV (yellow), V (Fuchsia), and VI (Aqua) colors in regular patterns.

**Figure 4:**
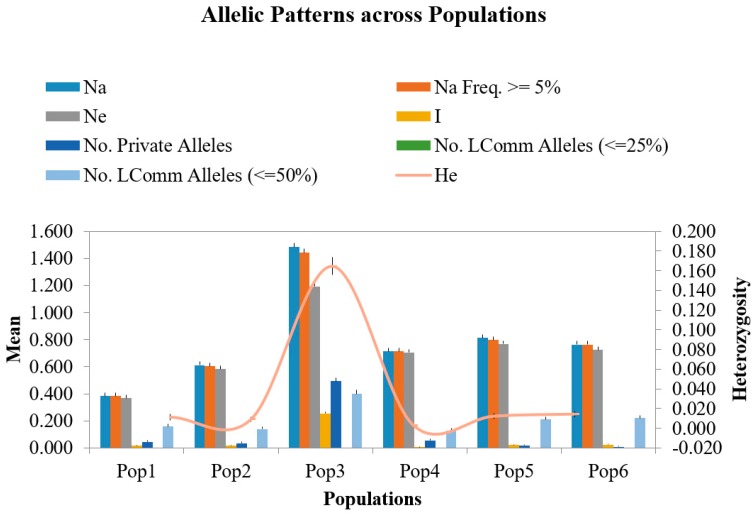
Alleleic patterns across populations.

Based on the ΔK value, the analysis of population structures divided 97 out of 132 accessions into six subpopulations (Figure 1, Figure 2 and Figure 3). Group 1 contained five diploid (D3) accessions collected from Santa Cruz Island. Group 2 had 32 accessions of *G. tomentosum* (AD3) obtained from a Hawaiian Island. Group 3 was composed of 14 accessions and was demarcated with the accession of *G. darwinii* (AD5) collected from Isabella Island but also including one from the China Wild Cotton Germplasm Nursery. Group 4 had 10 accessions of *G. ekmanianum* (AD6) collected from the Dominican Republic, NPGS, USA. Group 5 contained 19 accessions of *G. darwinii* that were collected from San Cristobal. Group 6 had 17 accessions of *G. barbadense* (AD2); of them, two were collected from the China Wild Cotton Germplasm Nursery (Supplementary Table S5). Based on a phylogenetic analysis using the Unweighted Pair-Group Method using Arithmetic average (UPGMA), the same accessions were placed under discriminating subgroups having significant genetic distance in accordance with the geographical locations of the collection. The results were further validated by using Shannon’s information index to determine the genetic diversity among six populations. It was found that population 3 (Isabella) had the highest degree of heterozygosity with 55.5% polymorphic loci (Figure 4).

### 2.5. Genetic Diversity and Cluster Analysis of Phylogenetic Tree

**Figure 5:**
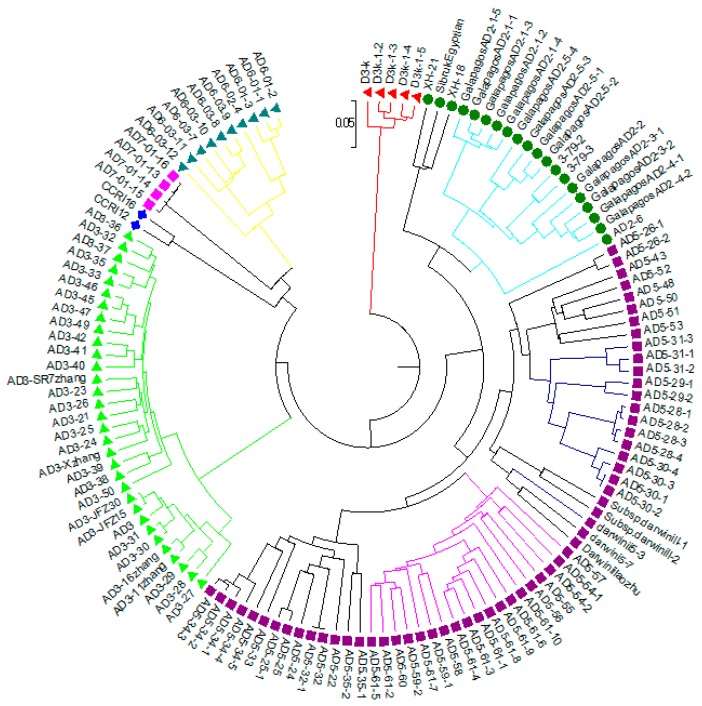
Dendrogram of 132 wild cotton accessions by Unweighted Pair-Group Method using Arithmetic average (UPGMA) analysis. Colors in the dendrogram lines correspond to *Gossypium* accession populations as identified by structure analysis while the colors in the circle represent the seven species. A membership threshold of 70% was used to assign accessions to different clusters in this dendrogram based on structure analysis.

**Figure 6:**
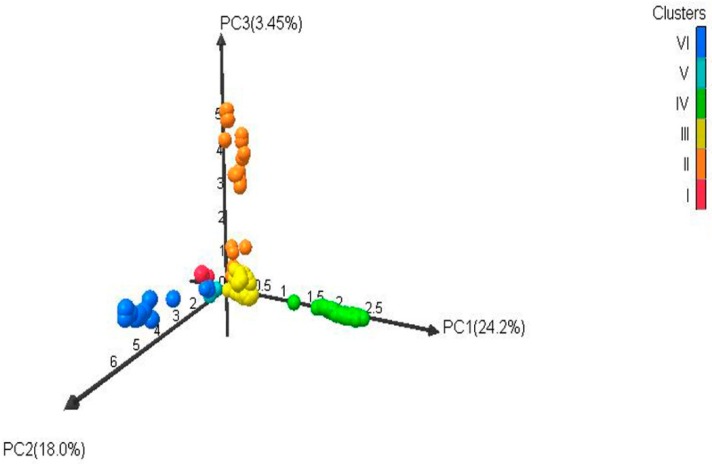
Three-dimensional principal coordinate analysis (PC_O_A) of a *Gossypium* accessions diversity panel genotyped with expressed sequence tags (EST) and Genomic simple sequence repeats (SSRs). The different colors in the figure correspond to six clusters: Red (Cluster I), orange (Cluster II), yellow (Cluster III), Bright green (Cluster IV), Sky blue (Cluster V), Blue (Cluster VI).

A total of 382 alleles, generated by 111 EST-SSRs, were used to run UPGMA for generating the dendrogram. Based on Nei’s criteria [52], the genetic distance among wild cotton accessions ranged from 0.003 to 0.529 with an average of 0.325. The highest genetic distance (0.529) was between D3k-21-3 and AD5-lz. The phylogenetic tree was in agreement with the structure results with the exception that *G. hirsutum* and *G. stephensii* sit in different clusters in the phylogenetic tree but in the structure analysis these were grouped together. In order to see how the results correspond to each other between the STRUCTURE and phylogenetic analyses, the dendrogram was manually edited to show the STRUCTURE grouping (Figure 5 and Supplementary Figure S1). Six groups identified in the structure analysis were also clustered together in the phylogenetic tree analysis. Overall, there was good agreement between the two estimates. The clustering pattern also showed agreement with relationships based on pedigree studies [53]. The first two axes of the principal coordinate analysis (PCoA) accounted for 42.2% of the variation (Figure 6). This indicates a high level of genetic diversity in the *Gossypium* germplasm with continuous variation between and within the subgroups. Analysis of molecular variance (AMOVA) revealed highly significant variation between the six groups identified by the structure analysis, with 49% of the total variation contributing to between-group differences. However, a larger amount of variation (51%) was due to diversity within the groups having different populations (Table 2). Pairwise F_ST_ analysis revealed that accessions from Pop 3 (Isabella region) are closer to accessions from the San Cristobal (Pop 5) and Santa Cruz regions (Pop 6) as compared with the Hawaiian accessions. The highest genetic differentiation was observed among tetraploid populations between accessions from the Hawaiian (Pop 2) and Santa Cruz (Pop 6) regions with a pairwise F_ST_ of 0.752 (*p* < 0.001) (Table 3).

A cluster analysis clearly discriminated diploid wild-type cotton from other tetraploid wild-types. These accessions were collected from different locations, namely the Galapagos Islands, Hawaii, the Dominican Republic, Wake Atoll, and the Wild Cotton Germplasm Nursery of China. The dendrogram was truncated at a genetic distance level of (0.05) and divided 132 cotton genotypes into seven clusters (Supplementary Figure S1).

### 2.6. Phylogenetic Analysis of Mined Genes and Functional Annotation of DUF819 (PF005684)

**Figure 7:**
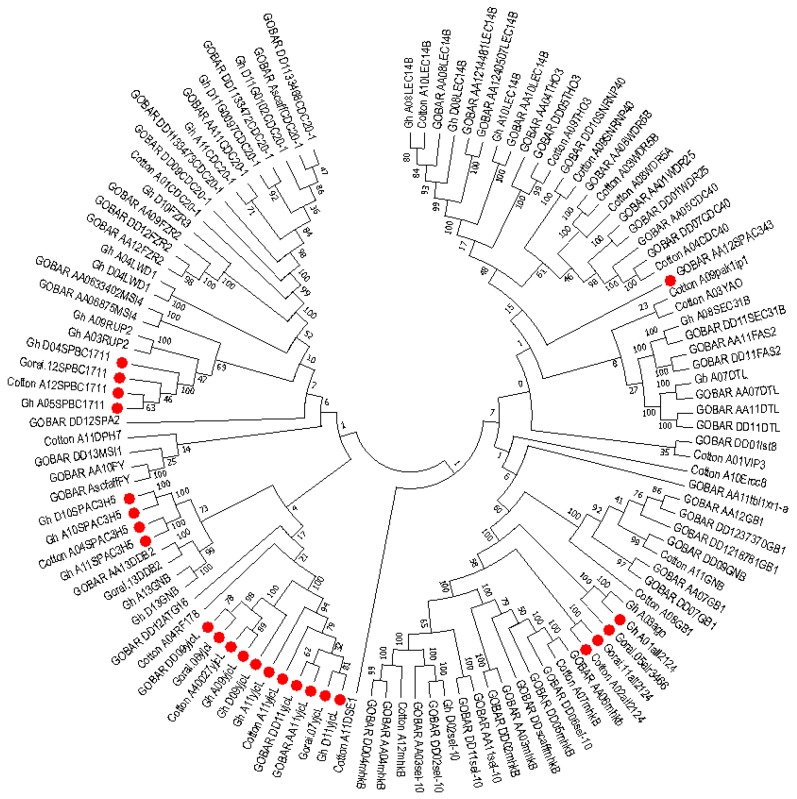
Evolutionary relationship of 115 genes belonging to protein domains of DUF819 (PF005684) and WDR (PF00400) in *Gossypium arboreum*, *G. raimondii, G. hirsutum,* and *G. barbadense.* The phylogenetic tree was constructed using MEGA software ver. 7.0 by the neighbor-joining method. The parameters were 1000 bootstraps and pairwise deletion. The 24 uncharacterized genes are indicated by red dots in four *Gossypium* species.

**Figure 8:**
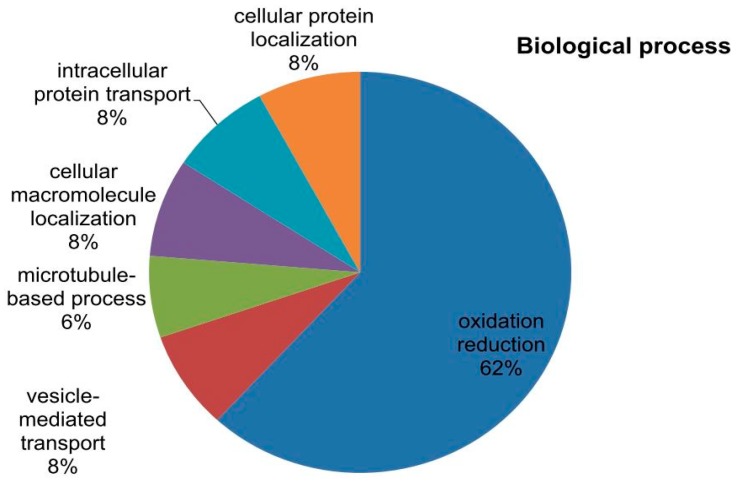

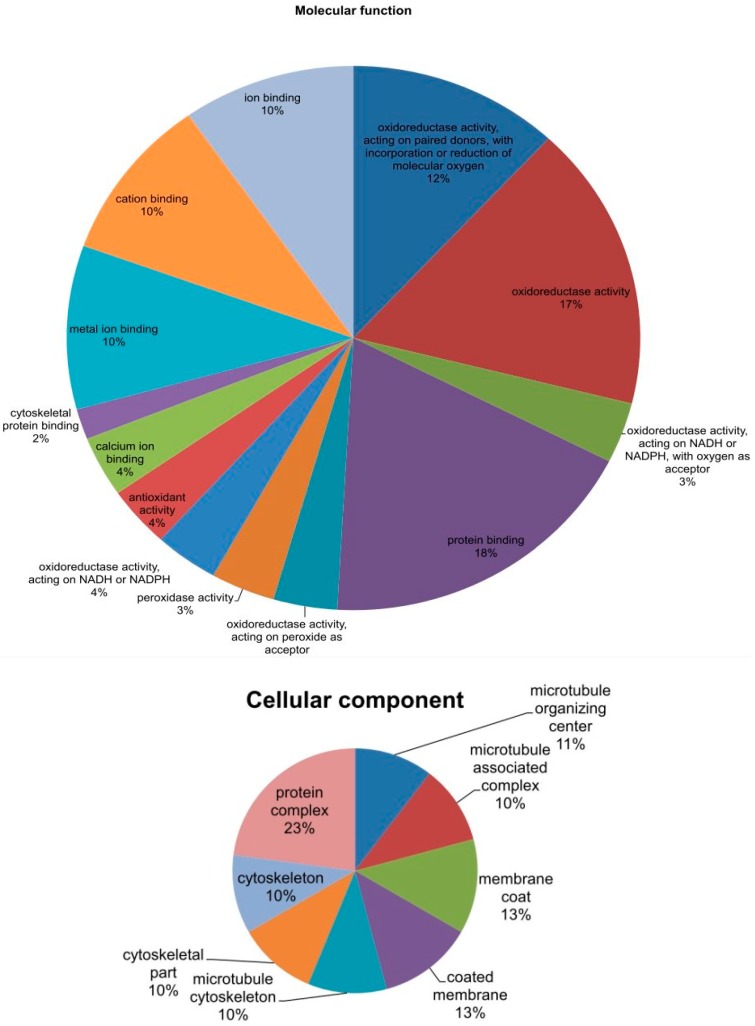
Genes were analyzed using the Agrigo v 2.0 software. Gene Ontology (GO) annotation results for *Gossypium hirsutum* mined genes. GO functional classification of genes mined with protein sequences predicted for their involvement in biological processes (BPs), molecular functions (MFs), and cellular component (CCs).

**Figure 9:**
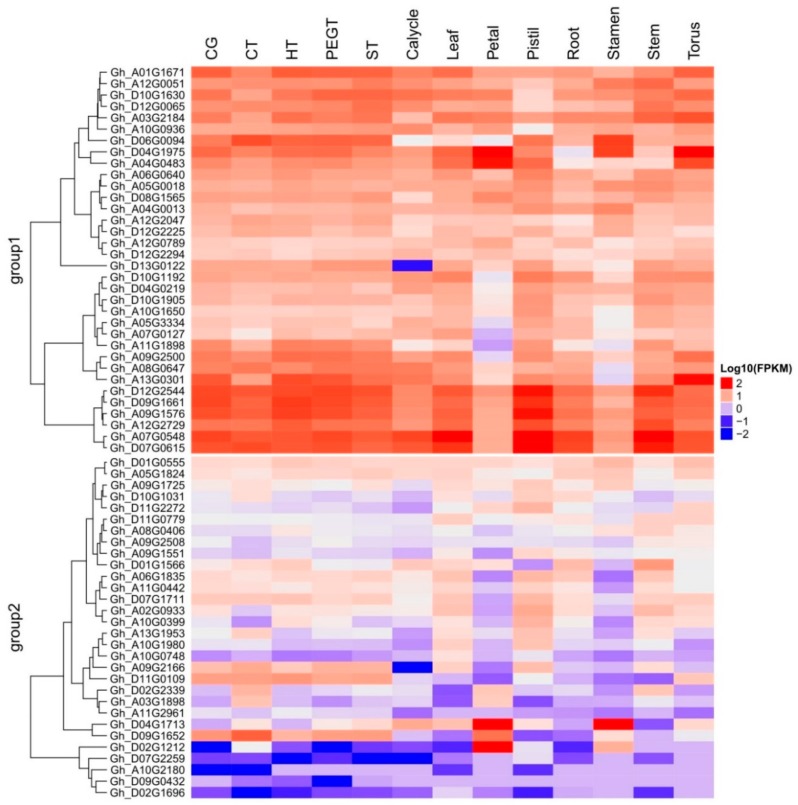
RNA sequence data analysis of 65 differentially expressed genes in eight different cotton tissues with reference to a control group (CG) under different cold (CT), heat (HT), PEG (PEGT), and salt treatments (ST) listed at the top of the figure. The names of genes are listed to the left of the figure. The heat map was generated from the log10 (FPKM) of the expression values by using R software. The *Y* axis represents the relative expression (2^−∆∆*C*t^).

The study was extended to dissect the evolutionary relationship among the uncharacterized genes because these are considered to be highly conserved and have an important role in biology. DUF819 is a family containing proteins (PF005684) found in 532 species with a total of 756 sequences. A total 1517 genes were found among *Gossypium arboreum* (258), *G. raimondii* (258), *G. hirsutum* (513), and *G. barbadense* (488) from the cotton functional genomic database (www.cottonfgd.org). These were the best-fit matched homologue genes having the highest similarity with the four cotton species. Out of 1517, only 116 genes differentially expressed in experiments and belonging to PF00400 and PF05684 were identified among *G. arboreum*, *G. raimondii*, *G. hirsutum,* and *G. barbadense.* A total of 24 uncharacterized genes identified to diagnose their evolutionary relationship with these cotton species. A phylogenetic tree consisting of 115 genes out of 116 expressed genes, including uncharacterized genes, in different experiments was constructed for the sorted PF00400 (105) and PF005684 (11) (Figure 7). The protein sequence of one gene remained unaligned during a ClustalW alignment. The PF00400 belonging to the superfamily WDR was used as a reference because 13 out of 24 uncharacterized genes were linked to this protein domain. The remaining 11 uncharacterized genes were named yjcL. The 11 yjcL (PF005684) genes were found to be more closely related to Cotton_A04_RF178 and then to GOBAR_DD_12SPA2, whose functions are known. Cotton_A04_RF178 is a well-known E3 ubiquitous protein having an important role in stress response in plants and animals [54]. It is predicted that, as these genes make a very close cluster with a well-known gene playing a crucial role in plant survival, they may have same function because they can be assigned to proteins by using the bioinformatics tools in comparative genomics [55]. The yjcL of A_ and D_, which are subgenomes of *G. hiursutum* and *G. barbadense*, make close groups with the yjcL of *G. arboreum* and *G. raimondii*, respectively. This indicates that yjcL genes may flow from *G. arboreum* and *G. raimondii* to *G. barbadense* and *G. hirsutum* in equal proportion. Moreover, the uncharacterized genes in *G. hirsutum*, *G. raimondii,* and *G. arboreum* indicated as all2124 are grouped close to Gh_A03ago, which has the known function of a protein related to F-box/WD repeats. Similarly, these results can also be validated by predicting that Gh_A01all2124, Gorai-all2124, Gorai-alr3466, and Cott_A_all2124 perform the same function as that determined for Gh_A03ago. GOBAR_AA12SPAC343 is separate from but close to the gene pak1ip1 with the known function of p21-activated protein kinase-interacting protein 1-like. The gene SPBC1711 located on the A_ and D_ genome of *G. hirsutum*, *G. arboreum*, and *G. raimondii* makes a close group with the gene named RUP2 in the A03 and A09 chromosomes of *G. hirsutum*. RUP2 is a gene composed of WD protein domains. Meanwhile, another gene, SPAC3H5, originating in *G. hirsutum* and *G. arboreum* was found to be lying nearest to the DDB2 gene of known function in *G. hirsutum*. These genes were distributed throughout the 26 chromosomes. The maximum number of genes (11) was found on chromosome D11, and the minimum number (4) was found on chromosome 2 (Supplementary Table S6). The coding DNA sequences (CDS) were characterized and the GC content percentage ranged from 38.9 to 52.7 with its length ranging from 417 to 3221 (bp). The maximum number of exons was noted to be 24 in the Gh_D11G0779 homologue of GOBAR_DD21377, indicating the highest intron disruption (Supplementary Table S6). The majority of these mined genes, especially the uncharacterized ones, have a single protein domain, which means that these genes are highly conserved. We analyzed the features of these genes and the results showed several categories related to stress and fiber development in upland cotton. We further analyzed the genes through annotations and Gene Ontology (GO) terms that were associated with the mined genes, which describe the genes in relation to cellular components (CCs), molecular function (MF), and biological process (BP) [56]. In cellular components, functions such as microtubule organizing center (11%), microtubule-associated complex (10%), membrane coat (13%), coated membrane (13%), cytoskeleton part (10%), microtubule cytoskeleton (%), cytoskeleton (10%), and protein complex (23%) were observed. Similarly, 14 molecular functions and 5 biological processes were observed (Figure 8). Finally, we carried out RNA sequence expression to validate our results. The 65 genes with differential expression in *G. hirsutum* were selected to construct a heat map. The genes were both up and downregulated in cold, hot, polyethylene glycol (PEG), and salt treatments and different developmental stages of different tissue organs, such as calyx, leaf, petal, pistil, root, stamen, stem, and torus tissue (Figure 9). The genes were categorized into two main groups. Group 1 comprised 34 genes that were significantly expressed; i.e., with fragments per kilobase of transcript per million mapped reads (FPKM) value of more than 1. Among the 34 upregulated genes, SPA2 (protein SPA1-RELATED 2) with Gene ID Gh_D12G2294 has five Go functions: protein kinase activity (GO:0004672 = MF), protein binding (GO:0005515 = MF), ATP binding (GO:0005524 = MF), protein phosphorylation (GO:0006468 = BP), and transferase activity transferring phosphorus-containing groups (GO:0016772 = MF). CDC40 (Pre-mRNA-processing factor 17) with Gene ID Gh_A05G0018 depicts three GO functions: mRNA splicing via spliceosome (GO:0000398 = BP), protein binding (GO:0005515 = MF), and catalytic step 2 spliceosome (GO:0071013 = CC). Two Guanine nucleotide-binding protein subunit beta-2s with different Gene IDs were found to have two similar GO functions, namely protein binding (GO: 0005515 = MF) and signal transduction (GO:0007165 = BP). All remaining genes were found to be associated in molecular function with protein binding with GO:0005515. The yjcL-linked gene ID Gh_A09G2500 showed significant expression against drought and salt stress and fell into group 1. The other two Gene IDs associated with SPAC3H5, an uncharacterized WD-repeat-containing protein (GO:0005515 = MF), also indicated significant expression. Group 2 has 31 genes that exhibited the differential expression of both up and downregulation (Supplementary Table S7). Among these, only the Gh_D07G1711 gene showed three GO functions: mRNA splicing via spliceosome (GO:0000398 = BP), protein binding (GO:0005515 = MF), and catalytic step 2 spliceosome (GO:0071013 = CC). All others were associated with WDR25 (WD-repeat containing protein 25) with the GO function GO:0005515 = MF except for three genes with the IDs Gh_D11G0109, Gh_A11G2961, and Gh_D09G0432, which are linked to the uncharacterized gene yjcL and have no GO functions. In the second group, four genes, namely Gh_D07G2259, Gh_A10G2180, Gh_D09G0432, and Gh_D02G1696, were relatively downregulated while all other genes showed differential expression. Gh_D04G1713 showed upregulated expression in petal and stamen tissues but relative downregulation in other tissues and under stress treatments.

## 3. Discussion

In this study, 111 SSR primer pairs generated 382 polymorphic loci in the 132 tested accessions. An average of 3.44 alleles amplified per marker was observed for all accessions, ranging from 2 to 8 alleles. This value is comparable with the findings of Dahab et al. [57] on allele number using 70 SSR markers on *Gossypium hirsutum*. Consistent results were found with 3.93 alleles per locus by Bardak and Bolek [58] for assessing genetic diversity in diploid and tetraploid cottons using Simple Sequence Repeat (SSR) and Inter Simple Sequence Repeat (ISSR) markers. Wendel and Percy [17] detected 3.47 enzymes per locus by studying 17 enzymes encoding 59 loci in a collection of 58 accessions of *darwinii* from six islands. However, other studies showed a variable allele number per locus. For example, 2.13, 2.20, 5.46, and 7.64 alleles per marker have been found in several genetic diversity assessments for the cotton germplasm [26,59,60,61]. The semi-wild accessions retaining a diverse germplasm showed high allele numbers in the majority of studies consistent with a recent study because these accessions have not yet been exposed to extensive human selection pressure for accumulating a particular type of alleles [26,62,63]. The number of alleles observed per marker is contingent on the selection of markers, the collection of germplasm to be genotyped, and the platform used for the resolution of amplified products [64].

Our study results determined an average PIC value of 0.555 with a range of 0.078 to 0.821, which completely corresponds to the literature-cited average PIC value for cotton SSRs, which ranges from 0.122 [65] to 0.71 [26]. Higher PIC values in cotton as shown in the current study suggest that these accessions can be useful for improving cotton [57]. The unique alleles identified in this study had percentages of 7.36, 5.83, 6.43, 17.36, 17.51, 3.2, and 14.44 in *G. barbadense*, *G. darwinii*, *G. tomentosum*, *G. hirsutum*, *G. ekmanianum*, *G. stephensii* and *G. klotzschianum*, respectively. These are higher than the percentages reported in the earlier report [65]. It is an interesting finding that all the unique alleles were found in the newly collected accessions along with a few from *G. hirsutum*. The unique alleles may be related to unique characteristics, such as extra-long fibers in *G. barbadense* and drought and salt tolerance in *G. tomentosum* and *G. darwinii*, and be similar to other wild accessions.

The common alleles were estimated to understand the gene flow mechanism of the *Gossypium* species during evolution. The results are supported by previous studies and the hypothesis that *G. hirsutum* has a single evolutionary lineage because all of the species in this study have common alleles with reference to the fixed alleles of *G. hirsutum*. *G. tomentosum* is considered to be a sister of *G. hirsutum*, while *G. barbadense* originates from a geographically overlapping region. *G. darwinii* has the same origin, the Galapagos Island, as *G. barbadense*. The *G. klotzschianum* diploid cotton is considered endemic to the New World and has an origin similar to that of *G. raimondii*. The two new species *G. ekmanianum* and *G. stephensii* are sister clades even though they make distinct groups with *G. hirsutum*, but *G. ekmanianum* and *G. stephensii* are monophyletic to *G. hirsutum* which strengthens the hypothesis of gene flow from different species to *G. hirsutum* having a single-lineage evolution [1,13,15]. The present analysis with a high level of natural introgression among the wild accessions shows consistency with the results of Yu et al. [51] and Hinze et al. [36] who described the distribution of introgression within the *G. hirsutum* and *G. barbadense* genomes using the chromosome positions as markers.

Populations from different islands are isolated distinctly, indicating general correspondence to Wedel and Percy’s investigations [17]. Due to good agreement with Wendel and Percy [17], an exploration that occurred 30 years prior to the present study, this novel study will also be helpful in understanding the basis of the hybridization and domestication phenomenon in cotton evolution. Our findings also suggest that a wild germplasm has higher genetic diversity than that in cultivated cotton.

A phylogenetic tree constructed based on genotypic data completely validated the distinct clustering of the accessions detected. The results are quite congruent to prior taxonomic studies [2,3,66]. The average genetic distance (GD = 0.325) revealed the overall level of genetic diversity to be high among semi-wild and cultivated accessions; this finding is similar to earlier reports [26,67,68]. However, this estimate may be inflated since data from monomorphic SSR loci were excluded in the current study.

An evolutionary relationship among the genes was also developed. It has been estimated that the majority of genes are linked to responses towards biotic and abiotic stress conditions. For example, the damage-specific DNA binding protein 2 (DDB2), Autophagy-related protein 16 (ATG16), WD repeat-containing protein LWD1, Denticleless protein homolog (DTL), Protein FIZZY-RELATED FZR2, FZR3, Pre-mRNA-processing factor 17 (CDC40), Diphthine methyltransferase (DPH7), Chromatin assembly factor 1 subunit FAS2, DNA excision repair protein ERCC-8, Flowering time control protein FY, Guanine nucleotide-binding protein subunit beta-like protein (GB1), Myosin heavy chain kinase B (mhkB), WD-40 repeat-containing protein MSI1, MSI4, F-box/WD repeat-containing protein sel-10, U5 small nuclear ribonucleoprotein 40 kDa protein (SNRNP40), THO complex subunit 3 (THO3), WD repeat-containing protein VIP3, WDR25, WDR5A, WDR5B, and U3 snoRNP-associated protein-like YAO belong to the superfamily of WDR and are involved in repairing damaged DNA under various stress conditions [69,70]. Similarly, RUP2 has been found to play a very crucial role in vegetative development and flowering in Arabidopsis [49]. F-box/WD repeat-containing protein 7, named as “ago” and associated with Gh_A03G1152, supports plants against disease and repairs damaged DNA [71]. SEC31 homolog B transports proteins and is situated at Golgi-associated endoplasmic reticulum exit sites. CDC20-1 is a known component of the anaphase promoting complex/cyclosome (APC/C), a cell-cycle-regulated E3 ubiquitin–protein ligase complex that controls progression through mitosis and the G1 phase of the cell cycle. This protein is involved in the pathway protein ubiquitination, which is part of protein modification. The intron-containing CDC20 gene copies provide conserved and redundant functions for cell-cycle progression in plants and are required for meristem maintenance, plant growth, and male gametophyte formation [69]. These results also support our hypothesis that yjcL (PF005684) has the same functions as these WDR family genes.

The current study is highly associated with the pedigree information recently provided by Gallagher et al. [3] after molecular confirmation of newly designated species of *Gossypium*. Genetic diversity within the group was highest for the Isabella group and lowest for the Hawaiian group (Table 2 and Figure 1). The genetic differentiation between groups was further validated by AMOVA, with 49% of the variation among populations and 51% of the variation within populations (** significant *p* > 0.001) being explained by the population structure of the wild cotton germplasm (Table 3). Such higher variation may be due to the complete study of seven different species of diploid and tetraploid cotton. This also indicates the presence of a great genetic difference among tetraploid and diploid cottons as well as a good level of genetic diversity within each group, which can be used in further hybridization breeding programs in cotton to broaden the narrow genetic base of *G. hirsutum*, which is becoming a serious threat due to limited allelic availability [72]. The F_ST_ values for the diploid and tetraploid cottons observed in this study (0.301–0.869) are very high, indicating high genetic distance and diversity. PCoA plots separated tetraploid cottons from diploid plants, supporting the AMOVA results. All these results are in good agreement with Noormohammadi et al.’s genetic diversity analysis [68] between diploid and tetraploid accessions.

Thus, our results could help breeders to determine the selection of appropriate parental combinations in germplasm enhancement programs and conserve genetic diversity and the evolutionary relationship among the genes of uncharacterized functions. The presence of profound population differentiation could pose a challenge to successful Genome-Wide Association Mapping (GWAS) studies in the Upland cotton germplasm for traits that are associated with population structures. The power of structure-based association studies to detect the effects of a single gene would be reduced if a large fraction of variation was explained by the population structures [22,73]. In such cases, alternative association mapping populations would be more useful.

## 4. Materials and Methods

This study was conducted at the Institute of Cotton Research (ICR, Anyang, China), Chinese Academy of agricultural Sciences (CAAS), Anyang, China. The cotton accessions were obtained from six islands, namely Santa Cruz, San Cristobal, Isabella of Galapagos Island, a Hawaiian Island, Dominican Republic, and Wake Atoll. The screening of this unique collection was carried out using microsatellite markers for the detection of a polymorphism among these accessions.

### 4.1. Plant Material and DNA Extraction

We sampled a total of 132 accessions belonging to different species, including five *G. klotzschianum* (D3), two *G. hirsutum* (AD1), 20 *G. barbadense* (AD2), 32 *G. tomentosum* (AD3), 59 *G. darwinii* (AD5), 10 *G. ekmanianum* (AD6), and four *G. stephensii* (AD7), from six islands and the Wild Cotton Germplasm Nursery of China. Among the 132 accessions, 32, 25, 16, 32, 10, 4, and 12 were obtained from Santa Cruz, Isabella, San Cristobal, a Hawaiian island, the Dominican Republic, Wake Atoll (NPGS, USA), and the Wild Cotton Germplasm Nursery of China, respectively (Supplementary Table S1). Seedlings of these accessions were grown at the wild cotton germplasm nursery of China, Sanya Hainan during October 2015, 2016, and 2017, respectively. When the plants were about 30–35 days old, fresh leaves were sampled and immediately frozen at −80 °C for later DNA extraction. Total genomic DNA was extracted from the frozen leaves by the cetyltrimethylamonium bromide (CTAB) method as described by Zhang and Stewart [74] with slight modifications. DNA was quantified using Nanodrop at a 260/280 nm absorbance ratio and the quality was checked by 1% (*w*/*v*) agarose gel electrophoresis.

### 4.2. SSR Marker Selection and Genotyping

A total of 853 randomly selected SSR markers, including 200 DPL, 310 MonCGR, 48 NAU, 41 MUCS, and 254 SWU, were surveyed for their polymorphisms in 132 genotypes belonging to seven cotton species. Then, 111 EST and genomic SSR (based on d-genome) polymorphic primers from the Cotton Marker Database (CMD; http://www.cottonmarker.org/) were used in the SSR analysis. The reaction contained 5 µL 2× Taq Master Mix (containing buffer, dNTPs, and Taq DNA Polymerase), 2 µL primers, 1 µL DNA, and 2 µL H_2_O. The PCR reaction was performed using a together TP 600 thermal cycler (TAKARA Bio Inc., Kusatsu, Japan) and then followed by silver staining according to a previous method described by Zhang et al. [75]. The PCR temperature program was two cycles of 95 °C for 3 min pre-denaturing followed by 30 cycles of 94 °C for 45 s denaturing, 57 °C for 36 s annealing, 72 °C for 1 min extension, with a final step of 1 cycle at 72 °C for 5 min extension. To confirm that the observed amplicons were amplified from genomic DNA and not a primer artifact, genome DNA was omitted from the control reaction. No amplification products were detected without genomic DNA in any PCR.

### 4.3. Analysis of Genotypic Data and Genetic Diversity

Pairwise genetic distances between accessions were calculated using the Powermarker software package ver.3.25 by Nei et al. [52] D_A_ distance. The dendrogram was constructed on the basis of the distance matrix. We estimated the similarity between genotypes for each accession by awarding a score to each microsatellite (i.e., 0 when an allele was absent, 1 when the allele was present). The cluster analysis was carried out using the unweighted pair group method using arithmetic average (UPGMA) and the dendrogram resulting from these calculations was plotted using MEGA 6.0 to visualize and edit the dendrogram. The basic summary statistics for biallelic data were calculated using the POWERMARKER software package version 3.25 [76]. The polymorphism information content (PIC) of an SSR marker was determined according to the method described by Anderson et al. [77] based on the allele frequency of all genotypes.

(1) $$\mathrm{PIC}\;=\;1\;-\;\sum_{i=1}^{n}pi2$$

where *P_ij_* is the frequency of the allele for locus *i* and the summation covered *n* patterns.

A PIC value of 1 indicates that the marker can differentiate each line, and 0 indicates a monomorphic marker. The informative potential of a marker is high if its PIC value is more than 0.5, moderate if its PIC is between 0.5 and 0.25, and only slightly informative if its PIC value is below 0.25. Other statistics calculated were the number of alleles and availability and gene diversity for each marker. Further analysis of genetic structure was done by means of Principal co-ordinate analysis (PC_O_A) using XLSTAT, 2014 [78] and a three-dimensional diagram was constructed. Dominant data (0, 1 binary data) were used for the PCoA analysis.

### 4.4. Analysis of Genetic Structure

The STRUCTURE software version 2.3.4 [79] was employed to define 132 accessions into clusters consisting of genotypes by using co-dominant genotypic data. The admixture model was used to estimate a mixed group by using correlated allele frequencies between populations as described by Falush et al. [80]. The optimum number of subpopulations was calculated based on the recommendation of Evanno et al. [81] by defining the values for *K* = 2 to *K* =10 with a burn length of 10,000 and a run length of 100,000 each in 10 runs. The results were uploaded in a Zip file to the STRUCTURE harvester software for finding the Δ*K* [82]. Grouping and subgrouping of accessions was done if the probability of membership was more than 70% [83]. The accessions with membership <70% were placed into the mixed subgroup.

### 4.5. Gene Mining and Phylogenetic Analysis of DUF819 Proteins

The complete sequence of Markers SWU15000–SWU15194 mapped for chromosome 6 was downloaded from the Genome database of *G. raimondii* [84] and blastx was used to find the homologue similarity of genes in the genome sequence of *G. raimondii*, *G. arboreum*, *G. hirsutum,* and *G. barbadense*. The mining of genes from the marker regions has been done extensively; see for instance Kirungu et al. [43]. Similarly, the same has been applied by Magwanga et al. [85]. The uncharacterized gene named yjcL of DUF819 (PF008654) was selected for the evolutionary study of genes in sequenced *Gossypium* species. The full-length sequences of DUF819 (PF005684) were downloaded from the pfam database (http://pfam.xfam.org/). The dendrogram was constructed by using Molecular Evolutionary Genetics version 7.0 [86]. The functional description related to domains of uncharacterized proteins has been predicted using the protein sequence of 116 genes downloaded from the Cotton Functional Genomics database (www.cottonfgd.org) [87]. The evolutionary relationship among all selected genes was summed up to provide a clear picture of functions with reference to upregulated genes of the superfamily WDR.

Thus, 115 genes out of 116 were grouped into 6 major clusters and their evolutionary history was inferred using the Neighbor-Joining method [88]. The optimal tree with the sum of branch length equal to 41.56 is shown. The percentage of replicate trees in which the associated taxa clustered together in the bootstrap test (1000 replicates) is shown next to the branches [89]. The tree is drawn to scale, with branch lengths in the same units as those of the evolutionary distances used to infer the phylogenetic tree. The evolutionary distances were computed using the Poisson correction method [90] and the units for the number of amino acid substitutions per site. The analysis involved 115 amino acid sequences. All ambiguous positions were removed for each sequence pair. There were a total of 1466 positions in the final dataset. Evolutionary analyses were conducted in MEGA7 (Figure 7).

The explored genes were analyzed for their gene features, protein characteristics, and RNA expression using the cotton functional genome database (https://cottonfgd.org/search/), While GO functional classification was done using Agrigo ver. 2.0 software acquiring *Gossypium hirsutum* as the reference genome. The analysis of RNA expression data inferred was then carried out to construct a heatmap using the R statistical software package.

## 5. Conclusions

SSR markers can be used to describe the degree of differentiation between populations and to control the conservation of genetic resources. The study concludes that the evaluated cotton accessions have a broad genetic basis. The recurrent use of these accessions as parents will produce significant results. The genetic diversity and evolutionary relationship recognized among the uncharacterized genes and population structures established in this study would be informative to select parental accessions for breeding and genetic analysis as well as for efficient management and conservation of allotetraploid cotton genetic diversity. We identified DUF819 (PF005684), which is not only highly conserved but also plays an important role against biotic and abiotic stress, among four sequenced cotton species by using the WDR (PF00400) superfamily as reference genome-sequenced proteins. Additionally, the current diversity panel of semi-wild cottons will be invaluable as a community resource for measuring linkage disequilibrium (LD) and for fine-scale mapping of traits through LD mapping or a Genome-Wide Association Study (GWAS) that can be streamlined for genomics-assisted plant breeding programs. Our findings suggest that allotetraploid cotton species, including *G. barbadense* (AD2), *G. tomentosum* (AD3), *G. darwinii* (AD5), *G. ekmanianum* (AD6), and *G. stephensii* (AD7), are a rich source for the creation of genetic diversity in upland cotton.

## Figures and Tables

**Table 1:** Summary of unique (present in one accession) and rare alleles (present in <5% accessions) observed in a combined Panel of 132 accessions.

Panel	Total Alleles	Total Lines	Unique Alleles	Rare Alleles (Freq < 5%)
Combined Panel	382	132	123 (32.19%)	108 (28.27%)
*Gossypium barbadense*	258	20	19 (7.36%)	12 (4.65%)
*Gossypium darwinii*	309	59	18 (5.83%)	57 (18.44%)
*Gossypium tomentosum*	205	32	13 (6.34%)	15 (7.31%)
*Gossypium hirsutum*	143	2	25 (17.48%)	22 (15.38%)
*Gossypium ekmanianum*	177	10	31 (17.51%)	2 (1.13%)
*Gossypium stephensii*	125	4	4 (3.2%)	0
*Gossypium klotzschianum*	90	5	13 (14.44%)	0

**Table 2:** Analysis of molecular variance for wild cotton accessions among and within six populations as identified by STRUCTURE.

Source of Variation	*df*	Sum of Squares	Mean Squares	Estimated Variation	Percentage of Variation
**Among Pops**	5	4201.563	840.313	38.134 **	49%
**Within Pops**	126	4932.945	39.150	39.150	51%
**Total**	131	9134.508		77.284	100%

(PhiPT < 0.493; ** significance at *p* < 0.001).

**Table 3:** Pairwise Fst estimates for the five groups corresponding to six regions of accession collections as identified by STRUCTURE.

Populations with Origin	Pop1 (Santa Cruz)	Pop2 (Hawaiian)	Pop3 (Isabella)	Pop4 (Dominican Republic)	Pop5 (San Cristobal)
**Pop2 (Hawaiian)**	0.869				
**Pop3 (Isabella)**	0.697	0.651			
**Pop4 (Dominican Republic)**	0.757	0.689	0.544		
**Pop5 (San Cristobal)**	0.810	0.749	0.301	0.638	
**Pop6 (Santa Cruz)**	0.813	0.752 **	0.413	0.626	0.517

(** significance at *p* < 0.001).

## Supplementary Materials

Supplementary materials can be found at http://www.mdpi.com/1422-0067/19/8/2401/s1.

## Abbreviations

PIC	Polymorphism information content
MAF	Major Allele frequency
GD	Genetic distance
Pop	population
AMOVA	Analysis of molecular variance
PCA	Principal coordinate analysis
PCR	Polymerase chain reaction
DNA	Deoxyribonucleic acid
NPGS	National Plant Germplasm System
EST	Expressed sequence tags
gSSR	Genomic SSR
SSR	Simple sequence repeats
RFLP	Restriction fragment polymorphism
RAPD	Random amplified polymorphic DNA
AFLP	Amplified fragment length polymorphism
SNP	Single nucleotide polymorphism
G	*Gossypium*
WCGN	Wild cotton Germplasm Nursery of China
