# Supplementary material for: Assessment of Genetic Diversity, Population Structure, and Evolutionary Relationship of Uncharacterized Genes in a Novel Germplasm Collection of Diploid and Allotetraploid Gossypium Accessions Using EST and Genomic SSR Markers
Source: Int J Mol Sci. 2018 Aug 14;19(8):2401. doi: 10.3390/ijms19082401 (PMC6121227; doi:10.3390/ijms19082401)
Supplement: Supplementary file 1 [file ijms-19-02401-s001.zip › ijms-326291-suppl-final/Supplementary Table S1.pdf]

Supplementary Table S1: Genotype name, pedigree and origin of selected genotypes

Sr. No.	Genotype	Accession No.	Origin	Sr. No.	Genotype	Accession No.	Origin
1	*G. barbadense*	3-79-2	Wild cotton germplasm nursery of China	67	*G.darwinii*	AD5-60	San Cristobal, Galapagos islands
2	*G. barbadense*	3-79-3	Wild cotton germplasm nursery of China	68	*G.darwinii*	AD5-61-1	San Cristobal, Galapagos islands
3	*G. barbadense*	AD2-1-1 GLPS	SantaCruz,Galapagos islands	69	*G.darwinii*	AD5-61-2	San Cristobal, Galapagos islands
4	*G. barbadense*	AD2-1-2 GLPS	SantaCruz,Galapagos islands	70	*G.darwinii*	AD5-61-3	San Cristobal, Galapagos islands
5	*G. barbadense*	AD2-1-3 GLPS	SantaCruz,Galapagos islands	71	*G.darwinii*	AD5-61-4	San Cristobal, Galapagos islands
6	*G. barbadense*	AD2-1-4 GLPS	SantaCruz,Galapagos islands	72	*G.darwinii*	AD5-61-5	San Cristobal, Galapagos islands
7	*G. barbadense*	AD2-1-5 GLPS	SantaCruz,Galapagos islands	73	*G.darwinii*	AD5-61-6	San Cristobal, Galapagos islands
8	*G.barbadense*	AD2-6	Santa Cruz, Galapagos islands	74	*G.darwinii*	AD5-61-7	San Cristobal, Galapagos islands
9	*G. barbadense*	AD2-2 GLPS	SantaCruz,Galapagos islands	75	*G.darwinii*	AD5-61-8	San Cristobal, Galapagos islands
10	*G. barbadense*	AD2-3-1 GLPS	SantaCruz,Galapagos islands	76	*G.darwinii*	AD5-61-9	San Cristobal, Galapagos islands
11	*G. barbadense*	AD2-3-2 GLPS	Santa Cruz, Galapagos islands	77	*G.darwinii*	AD5-61-10	San Cristobal, Galapagos islands
12	*G. barbadense*	AD2-4-1 GLPS	Santa Cruz, Galapagos islands	78	*G. darwinii*	Subsp.darwini II-1	Wild cotton germplasm nursery of China
13	*G. barbadense*	AD2-4-2 GLPS	Santa Cruz, Galapagos islands	79	*G. darwinii*	Subsp.darwini II-2	Wild cotton germplasm nursery of China
14	*G. barbadense*	AD2-5-1 GLPS	Santa Cruz, Galapagos islands	80	*G. klotzschianum*	D3-k-lz	Wild cotton germplasm nursery of China
15	*G. barbadense*	AD2-5-2 GLPS	Santa Cruz, Galapagos islands	81	*G. klotzschianum*	D3k-21-1	Santa Cruz, Galapagos islands
16	*G. barbadense*	AD2-5-3 GLPS	Santa Cruz, Galapagos islands	82	*G. klotzschianum*	D3k-21-2	Santa Cruz, Galapagos islands
17	*G. barbadense*	AD2-5-4 GLPS	Santa Cruz, Galapagos islands	83	*G. klotzschianum*	D3k-21-3	Santa Cruz, Galapagos islands
18	*G. barbadense*	XH-18	Wild cotton germplasm nursery of China	84	*G. klotzschianum*	D3k-21-4	Santa Cruz, Galapagos islands
19	*G. barbadense*	XH-21	Wild cotton germplasm nursery of China	85	*G.hirsutum*	CCRI12	Wild cotton germplasm nursery of China
20	*G. barbadense*	Sibruk Egyptian	Wild cotton germplasm nursery of China	86	*G.hirsutum*	CCRI16	Wild cotton germplasm nursery of China
21	*G. darwinii*	AD5-lz	Wild cotton germplasm nursery of China	87	*G.tomentosum*	AD3-27	Hawaiian Islands
22	*G. darwinii*	AD5-3	Wild cotton germplasm nursery of China	88	*G.tomentosum*	AD3-28-1	Hawaiian Islands
23	*G. darwinii*	AD5-7	Wild cotton germplasm nursery of China	89	*G.tomentosum*	AD3-28-2	Hawaiian Islands
24	*G. darwinii*	AD5-25-1	SantaCruz,Galapagos islands	90	*G.tomentosum*	AD3-30-1	Hawaiian Islands
25	*G. darwinii*	AD5-26-1	Isabela, Galapagos islands	91	*G.tomentosum*	AD3-30-2	Hawaiian Islands
26	*G. darwinii*	AD5-26-2	Isabela, Galapagos islands	92	*G.tomentosum*	AD3-lz	Hawaiian Islands
27	*G. darwinii*	AD5-28-1	Isabela, Galapagos islands	93	*G.tomentosum*	AD3-70	Hawaiian Islands
28	*G. darwinii*	AD5-28-2	Isabela, Galapagos islands	94	*G.tomentosum*	AD3-71	Hawaiian Islands
29	*G. darwinii*	AD5-28-3	Isabela, Galapagos islands	95	*G.tomentosum*	AD3-72	Hawaiian Islands
30	*G. darwinii*	AD5-28-4	Isabela, Galapagos islands	96	*G.tomentosum*	AD3-73	Hawaiian Islands
31	*G. darwinii*	AD5-29-1	Isabela, Galapagos islands	97	*G.tomentosum*	AD3-68	Hawaiian Islands
32	*G. darwinii*	AD5-29-2	Isabela, Galapagos islands	98	*G.tomentosum*	AD3-69	Hawaiian Islands
33	*G. darwinii*	AD5-30-1	Isabela, Galapagos islands	99	*G.tomentosum*	AD3-21	Oahu, Hawaiian Islands
34	*G. darwinii*	AD5-30-2	Isabela, Galapagos islands	100	*G.tomentosum*	AD3-23	Oahu, Hawaiian Islands
35	*G. darwinii*	AD5-30-3	Isabela, Galapagos islands	101	*G.tomentosum*	AD3-24	Oahu, Hawaiian Islands
36	*G. darwinii*	AD5-30-4	Isabela, Galapagos islands	102	*G.tomentosum*	AD3-25	Oahu, Hawaiian Islands
37	*G. darwinii*	AD5-31-1	Isabela, Galapagos islands	103	*G.tomentosum*	AD3-26	Oahu, Hawaiian Islands
38	*G. darwinii*	AD5-31-2	Isabela, Galapagos islands	104	*G.tomentosum*	AD3-32	Lanai , Hawaiian Islands
39	*G. darwinii*	AD5-31-3	Isabela, Galapagos islands	105	*G.tomentosum*	AD3-33	Oahu, Hawaiian Islands
40	*G.darwinii*	AD5-43	Isabela, Galapagos islands	106	*G.tomentosum*	AD3-35	Big island, Hawaiian Islands
41	*G.darwinii*	AD5-48	Isabela, Galapagos islands	107	*G.tomentosum*	AD3-36	Big island, Hawaiian Islands
42	*G.darwinii*	AD5-50	Isabela, Galapagos islands	108	*G.tomentosum*	AD3-37	Lanai , Hawaiian Islands
43	*G.darwinii*	AD5-51	Isabela, Galapagos islands	109	*G.tomentosum*	AD3-38	Lanai , Hawaiian Islands
44	*G.darwinii*	AD5-52	Isabela, Galapagos islands	110	*G.tomentosum*	AD3-39	Lanai , Hawaiian Islands
45	*G.darwinii*	AD5-53	Isabela, Galapagos islands	111	*G.tomentosum*	AD3-40	Lanai , Hawaiian Islands
46	*G.darwinii*	AD5-54-1	Isabela, Galapagos islands	112	*G.tomentosum*	AD3-41	Lanai , Hawaiian Islands
47	*G.darwinii*	AD5-54-2	Isabela, Galapagos islands	113	*G.tomentosum*	AD3-42	Lanai , Hawaiian Islands
48	*G.darwinii*	AD5-55	Isabela, Galapagos islands	114	*G.tomentosum*	AD3-45	Molokai , Hawaiian Island
49	*G. darwinii*	AD5-22	SantaCruz,Galapagos islands	115	*G.tomentosum*	AD3-46	Molokai , Hawaiian Island
50	*G. darwinii*	AD5-24	SantaCruz,Galapagos islands	116	*G.tomentosum*	AD3-47	Molokai , Hawaiian Island
51	*G. darwinii*	AD5-25-2	SantaCruz,Galapagos islands	117	*G.tomentosum*	AD3-49	Molokai , Hawaiian Island
52	*G. darwinii*	AD5-32-1	SantaCruz,Galapagos islands	118	*G.tomentosum*	AD3-50	Oahu, Hawaiian Islands
53	*G. darwinii*	AD5-32-2	SantaCruz,Galapagos islands	119	*G.ekmanianum*	AD6-01-1	Dominican Republic, US NPGS
54	*G. darwinii*	AD5-33	SantaCruz,Galapagos islands	120	*G.ekmaianum*	AD6-01-2	Dominican Republic, US NPGS
55	*G. darwinii*	AD5-34-1	SantaCruz,Galapagos islands	121	*G.ekmanianum*	AD6-01-3	Dominican Republic, US NPGS
56	*G. darwinii*	AD5-34-2	SantaCruz,Galapagos islands	122	*G.ekmanianum*	AD6-02-4	Dominican Republic, US NPGS
57	*G. darwinii*	AD5-34-3	SantaCruz,Galapagos islands	123	*G.ekmanianum*	AD6-03-7	Dominican Republic, US NPGS
58	*G. darwinii*	AD5-34-4	SantaCruz,Galapagos islands	124	*G.ekmanianum*	AD6-03-8	Dominican Republic, US NPGS
59	*G. darwinii*	AD5-34-5	SantaCruz,Galapagos islands	125	*G.ekmanianum*	AD6-03-9	Dominican Republic, US NPGS
60	*G. darwinii*	AD5-35-1	SantaCruz,Galapagos islands	126	*G.ekmanianum*	AD6-03-10	Dominican Republic, US NPGS
61	*G. darwinii*	AD5-35-2	SantaCruz,Galapagos islands	127	G.ekmanianum	AD6-03-11	Dominican Republic, US NPGS
62	*G.darwinii*	AD5-56	San Cristobal, Galapagos islands	128	*G.ekmanianum*	AD6-03-12	Dominican Republic, US NPGS
63	*G.darwinii*	AD5-57	San Cristobal, Galapagos islands	129	*G.stephensii*	AD7-01-13	Wake Atoll, US NPGS
64	*G.darwinii*	AD5-58	San Cristobal, Galapagos islands	130	*G.stephensii*	AD7-01-14	Wake Atoll, US NPGS
65	*G.darwinii*	AD5-59-1	San Cristobal, Galapagos islands	131	*G.stephansii*	AD7-01-15	Wake Atoll, US NPGS
66	*G.darwinii*	AD5-59-2	San Cristobal, Galapagos islands	132	*G.stephansii*	AD7-01-16	Wake Atoll, US NPGS
