# Supplementary material for: Assessment of Genetic Diversity, Population Structure, and Evolutionary Relationship of Uncharacterized Genes in a Novel Germplasm Collection of Diploid and Allotetraploid Gossypium Accessions Using EST and Genomic SSR Markers
Source: Int J Mol Sci. 2018 Aug 14;19(8):2401. doi: 10.3390/ijms19082401 (PMC6121227; doi:10.3390/ijms19082401)
Supplement: Supplementary file 1 [file ijms-19-02401-s001.zip › ijms-326291-suppl-final/Supplementary Table S2.pdf]

Supplementary Table S2: SSR markers with repeat type, Number of Polymorphic alleles, Total alleles, major allele frequency (MAF), No. of obs. and polymorphism information content (PIC) estimated by 111 simple sequence repeat markers in 132 diploid and allotetraploid *Gossypium* accessions.

Marker	Repeat Type	Poly Alleles	Total bands	MAF	No. of obs.	PIC
DPL0064		3	54	0.863	131	0.547
DPL0068	(AG)27(GGGA)2	6	228	0.710	131	0.808
DPL0069	(AG)27	3	131	0.776	131	0.491
DPL0071	(AG)32	3	161	0.634	131	0.640
DPL0094	(AG)29	3	191	0.812	131	0.508
DPL0169	(TC)14	3	198	0.545	131	0.663
DPL0249	(TCA)8	7	247	0.828	132	0.713
DPL0262	(ATG)13	4	106	0.929	126	0.143
DPL0278	(CAT)8	5	304	0.779	128	0.721
DPL0279	(TCA)8 (CCA)4	3	138	0.697	132	0.568
DPL0300	(ATC)10	6	265	0.817	132	0.676
DPL0307	(GAT)7	2	228	0.898	127	0.499
DPL0309	(GAT)7	2	129	0.542	119	0.499
DPL0317	(TCTTCA)4(TCA)6(ADAAC)2	4	185	0.697	127	0.694
DPL0325	(TTC)14(ATC)11	3	112	0.861	127	0.296
DPL0328	(CAT)8	3	171	0.864	127	0.433
DPL0338	(CAT)8	3	136	0.682	127	0.525
DPL0342	(ATC)10	3	161	0.697	131	0.549
DPL0348	(GCT)8	2	147	0.674	132	0.457
DPL0368	(CAG)6(CAG)4(GCA)2(CAA)2(CAT)2(CAG)2(TTGCAG)3(CAG)3(CAA)4(GCA)4(GCA)2(ACA)2(GCA)5(CAG)7	5	223	0.777	132	0.683
DPL0378	(TAG)14	6	220	0.808	132	0.686
DPL0385	(TAG)9	6	179	0.870	132	0.612
DPL0389	(AGT)15(AAT)5(AAC)4	4	263	0.714	132	0.681
DPL0395	(TTA)13	6	240	0.710	132	0.794
DPL0417	(TAA)19	3	246	0.667	132	0.624
DPL0442	(ATT)12	4	126	0.759	131	0.747
DPL0444	(AAT)10	3	140	0.672	132	0.538
DPL0489	(ATAC)10	5	152	0.764	129	0.750
DPL0490	(ATGT)9	2	118	0.759	129	0.344
DPL0501	(TATG)11	8	334	0.748	126	0.821
DPL0513	(GTAT)21	4	109	0.789	123	0.597
DPL0608	(AAAAAG)2(GA)3(AG)20	5	231	0.782	125	0.695
DPL0622		2	130	0.641	128	0.462
DPL0631	(TCT)8	3	182	0.734	129	0.582
DPL0635	(CTT)23	2	77	0.703	130	0.385
DPL0638		3	130	0.724	129	0.545
DPL0639		3	182	0.584	125	0.653
DPL0651		2	155	0.760	129	0.407
DPL0679	(AGA)6	2	97	0.622	129	0.449

DPL0707		4	182	0.631	124	0.734
DPL0715	(TTG)7	3	147	0.886	129	0.343
DPL0727		4	116	0.862	129	0.407
DPL0732		4	199	0.775	129	0.620
DPL0788		3	179	0.544	129	0.661
DPL0807	(TCA)6(ATC)3(TCA)2(TCG)9	7	208	0.793	130	0.743
DPL0810	(TATC)11(TATC)4(CATC)5(TATC)2	4	171	0.666	128	0.725
DPL0852		2	163	0.721	129	0.439
MonCGR6280		3	170	0.855	131	0.445
MonCGR6294		2	106	0.703	125	0.389
MonCGR6382		2	166	0.779	129	0.406
MonCGR6407		3	133	0.680	129	0.533
MonCGR6512		2	114	0.762	126	0.332
MonCGR6517		2	68	0.780	93	0.208
MUCS0064	(CAG)4(GAG)6	4	229	0.781	129	0.633
NAU1028	(AATT)5	3	182	0.720	126	0.565
NAU1052	(AGT)6	2	87	0.775	129	0.167
SWU10037	(GAA)9	3	179	0.773	129	0.529
SWU10072	(CT)14	2	154	0.678	129	0.455
SWU10113	(TATG)11	3	117	0.698	129	0.652
SWU10116	(GGAGAG)4	3	197	0.558	128	0.662
SWU10326	(AC)15	2	164	0.828	131	0.363
SWU10332	(TTC)10	6	111	0.857	129	0.716
SWU10608	(TTC)11	4	171	0.674	131	0.716
SWU10663	(ATT)14	3	114	0.710	131	0.533
SWU10664	(TTA)13	2	107	0.599	126	0.473
SWU10718	(TTTTA)5	2	93	0.631	126	0.458
SWU10722	(TGTA)17	3	126	0.677	127	0.569
SWU10741	(AACA)5	3	136	0.760	129	0.533
SWU10746	(ACC)8	2	190	0.736	129	0.500
SWU10755	(TAGA)5	2	98	0.798	131	0.183
SWU10828	(ATGT)6	3	184	0.584	129	0.654
SWU10840	(TGA)10	3	66	0.829	129	0.506
SWU10892	(TTA)13	3	141	0.686	123	0.541
SWU10931	(GCAATG)4	2	212	0.828	128	0.499
SWU11097	(ATACAT)7	3	190	0.788	126	0.537
SWU11112	(CA)18-(TCTATA)7	3	249	0.747	128	0.631
SWU11115	(TGA)10	3	189	0.541	130	0.664
SWU11731	(GTT)7	3	194	0.690	131	0.604
SWU12187	(TGC)6	3	132	0.656	128	0.587
SWU12228	(AAAGAG)4	2	89	0.652	128	0.482
SWU12248	(TTC)17	2	71	0.723	128	0.499
SWU12430	(CATGCT)4	3	110	0.730	127	0.513
SWU12746	(GGA)10	2	93	0.746	124	0.285
SWU13423	(TC)14	2	126	0.656	127	0.450
SWU13534	(AGACA)4	2	139	0.707	128	0.427
SWU13551	(CCTAC)4	3	174	0.578	128	0.654
SWU13677	(AATGAG)6	4	170	0.708	132	0.650
SWU13893	(CATA)9	4	204	0.621	132	0.731
SWU13895	(ATAC)8	4	122	0.767	131	0.675
SWU14260	(GAT)17	4	88	0.827	127	0.572
SWU14461	(TTC)15	3	125	0.669	118	0.549

SWU14590	(CT)10	3	207	0.831	132	0.523
SWU14654	(GGAATA)5	3	164	0.672	132	0.620
SWU14702	(TA)12	2	196	0.784	125	0.495
SWU14818	(CAG)8	3	112	0.889	132	0.197
SWU14961	(TTGGCA)5	4	109	0.784	124	0.542
SWU15000	(TG)12	4	289	0.778	132	0.673
SWU15194	(ACAT)14	7	200	0.830	132	0.726
SWU15212	(CATA)6	3	222	0.886	128	0.522
SWU15473	(GAA)11	4	197	0.725	128	0.652
SWU15517	(ATAC)7	5	131	0.798	130	0.698
SWU15642	(AGTAT)6	5	173	0.738	132	0.773
SWU16339	(CT)10	3	222	0.569	130	0.661
SWU16373	(TG)10	4	169	0.680	132	0.706
SWU16441	(CAT)7	4	156	0.814	132	0.561
SWU16671	(TA)18	4	173	0.787	128	0.564
SWU16802	(GAA)7-(GAA)16	5	125	0.856	124	0.552
SWU18196	(GTTTG)6	5	248	0.689	131	0.739
SWU18252	(CAA)8	3	124	0.959	130	0.078
SWU18262	(ATATGT)8	5	128	0.802	129	0.725
SWU18289	(ACAT)6-(AT)12	5	136	0.784	126	0.700
**G Mean**		**3.441**	**161.73**	**0.738**	**128.432**	**0.555**
