# Supplementary material for: Assessment of Genetic Diversity, Population Structure, and Evolutionary Relationship of Uncharacterized Genes in a Novel Germplasm Collection of Diploid and Allotetraploid Gossypium Accessions Using EST and Genomic SSR Markers
Source: Int J Mol Sci. 2018 Aug 14;19(8):2401. doi: 10.3390/ijms19082401 (PMC6121227; doi:10.3390/ijms19082401)
Supplement: Supplementary file 1 [file ijms-19-02401-s001.zip › ijms-326291-suppl-final/Supplementary Table S3.pdf]

Supplementary Table S3: List of unique alleles found in allotetraploid accessions.

Sr.No.	Accession Name	Species Name	Unique Alleles
1	CCRI12	*G. hirsutum*	15
2	CCRI16	*G. hirsutum*	10
3	AD2-1 GLPS	*G.barbadense*	1
4	AD2-2 GLPS	*G.barbadense*	4
5	AD2-5 GLPS	*G.barbadense*	2
6	AD2-6	*G.barbadense*	4
7	3-79-2	*G.barbadense*	1
8	XH21	*G.barbadense*	6
9	Sibruk Egyptian	*G.barbadense*	1
10	AD3-27	*G.tomentosum*	4
11	AD3-28-1	*G.tomentosum*	1
12	AD3-28-2	*G.tomentosum*	1
13	AD3-40	*G.tomentosum*	1
14	AD3-47	*G.tomentosum*	1
15	AD3-49	*G.tomentosum*	2
16	AD3-50	*G.tomentosum*	1
17	AD3-69	*G.tomentosum*	2
18	AD5-22	*G.darwinii*	1
19	AD5-34	*G.darwinii*	1
20	AD5-43	*G.darwinii*	1
21	AD5-48	*G.darwinii*	2
22	AD5-54	*G.darwinii*	1
23	AD5-56	*G.darwinii*	2
24	AD5-57	*G.darwinii*	1
25	AD5-60	*G.darwinii*	1
26	AD5-61-9	*G.darwinii*	2
27	AD5-7	*G.darwinii*	2
28	AD5-3	*G.darwinii*	2
29	Subsp darwiniiII-2	*G.darwinii*	2
30	AD6-02-4	*G.ekmanianum*	5
31	AD6-03-10	*G. ekmanianum*	2
32	AD6-03-12	*G. ekmanianum*	24
33	AD7-01-13	*G. stephensii*	1
34	AD7-01-14	*G. stephensii*	1
35	AD7-01-15	*G. stephensii*	2
36	D3-k-lz	*G. klotzschianum*	6
37	D3-k-21-1	*G. klotzschianum*	2
38	D3-k-21-4	*G. klotzschianum*	5
	Total		**123**
