# Supplementary material for: Assessment of Genetic Diversity, Population Structure, and Evolutionary Relationship of Uncharacterized Genes in a Novel Germplasm Collection of Diploid and Allotetraploid Gossypium Accessions Using EST and Genomic SSR Markers
Source: Int J Mol Sci. 2018 Aug 14;19(8):2401. doi: 10.3390/ijms19082401 (PMC6121227; doi:10.3390/ijms19082401)
Supplement: Supplementary file 1 [file ijms-19-02401-s001.zip › ijms-326291-suppl-final/Supplementary Table S4.pdf]

Supplementary Table S5: Estimation of common alleles distributed among all six *Gossypium* species with reference to *G. hirsutum*.

Marker name	*G.bar* (AD2)	*G.darwinii* (AD5)	*G.tom* (AD3)	*G.ekma* (AD6)	*G.steph* (AD7)	*G.klotz* (D3)	Common Alleles
DPL0068-B	18	10	20	0	0	0	48
DPL0068-E	5	5	10	0	0	0	20
DPL0068-F	19	16	11	0	0	0	46
DPL0069-C	0	0	0	10	0	0	10
DPL0071-A	9	17	32	10	4	0	72
DPL0071-B	14	33	0	0	0	0	47
DPL0094-A	20	58	30	10	4	0	122
DPL0169-A	20	0	29	0	4	5	58
DPL0249-C	1	0	0	0	0	2	3
DPL0262-C	20	45	31	0	0	0	96
DPL0278-A	20	49	21	6	4	5	105
DPL0278-C	18	39	23	4	4	0	88
DPL0300-A	20	59	32	9	4	0	124
DPL0300-B	0	2	1	10	0	5	18
DPL0307-B	19	51	32	10	4	0	116
DPL0309-A	9	41	0	10	0	0	60
DPL0309-B	14	46	0	1	4	0	65
DPL0317-B	8	56	0	9	4	0	77
DPL0317-C	1	3	0	10	4	3	21
DPL0325-C	0	1	0	4	4	0	9
DPL0328-B	19	56	32	10	4	0	121
DPL0328-C	0	0	0	10	0	0	10
DPL0348-A	20	32	32	7	1	1	93
DPL0348-B	0	38	2	2	3	5	50
DPL0368-C	0	2	0	0	0	5	7
DPL0385-D	3	13	1	1	0	0	18
DPL0385-F	0	0	0	4	4	0	8
DPL0389-B	17	52	32	10	4	0	115
DPL0389-D	1	20	0	10	0	5	36
DPL0417-A	20	55	32	10	4	0	121
DPL0417-C	3	25	31	0	0	5	64
DPL0444-B	1	24	32	3	4	5	69
DPL0489-C	0	15	30	0	0	0	45
DPL0501-B	17	26	0	4	0	0	47
DPL0501-C	17	15	32	6	4	5	79
DPL0608-A	20	42	32	10	4	0	108
DPL0608-E	0	0	29	6	0	0	35
DPL0622-A	3	20	17	1	0	4	45

DPL0631-B	0	20	1	10	4	1	36
DPL0631-C	0	22	12	0	0	4	38
DPL0635-A	3	6	32	10	4	0	55
DPL0638-C	0	0	0	10	0	0	10
DPL0651-B	20	43	32	10	4	0	109
DPL0707-B	0	33	20	1	0	0	54
DPL0727-C	0	0	0	5	4	0	9
DPL0732-A	20	47	27	8	0	2	104
DPL0732-C	17	1	0	10	4	0	32
DPL0807-B	0	0	0	9	0	0	9
DPL0852-A	17	55	22	10	4	0	108
MonCGR6280-A	19	55	32	10	4	0	120
MonCGR6294-A	20	47	1	4	4	0	76
MonCGR6382-B	0	0	31	10	4	0	45
MonCGR6407-C	4	21	32	6	4	0	67
MonCGR6512-A	0	0	3	10	4	5	22
MonCGR6512-B	19	36	28	1	4	0	88
MUCS0064-C	0	8	18	0	0	0	26
NAU1028-B	1	51	32	10	4	0	98
NAU1028-C	1	26	30	1	4	0	62
NAU1052-A	20	47	6	4	0	0	77
SWU10037-A	16	42	32	9	4	5	108
SWU10037-B	6	33	1	10	0	0	50
SWU10072-B	17	34	32	6	4	5	98
SWU10116-A	16	9	31	10	4	0	70
SWU10116-C	16	7	32	9	4	0	68
SWU10326-A	20	53	31	10	4	5	123
SWU10326-B	3	19	5	10	0	0	37
SWU10663-C	17	20	4	8	4	0	53
SWU10664-A	1	24	29	6	4	0	64
SWU10722-C	2	28	8	3	4	3	48
SWU10741-C	0	13	0	10	0	0	23
SWU10746-A	19	33	31	9	4	0	96
SWU10746-B	18	30	31	2	4	5	90
SWU10755-A	17	55	0	10	4	0	86
SWU10828-B	2	11	32	10	4	0	59
SWU10828-C	3	33	0	9	0	0	45
SWU10892-C	0	0	0	1	4	0	5
SWU10931-A	18	54	32	1	4	0	109
SWU10931-B	18	36	31	10	4	0	99
SWU11097-A	16	38	32	10	4	0	100
SWU11112-B	0	10	21	8	4	0	43
SWU11112-C	19	39	30	9	4	3	104
SWU11115-A	18	24	3	10	0	0	55

SWU11731-C	7	53	6	0	4	1	71
SWU12187-B	20	3	31	7	0	0	61
SWU12228-B	19	0	10	0	0	5	34
SWU12248-A	0	25	0	0	4	3	32
SWU12248-B	0	12	19	0	0	4	35
SWU12430-B	0	51	3	7	4	0	65
SWU12430-C	20	7	8	0	0	0	35
SWU12746-B	14	46	10	5	0	0	75
SWU13423-B	19	32	22	4	4	0	81
SWU13534-B	20	23	32	10	4	5	94
SWU13551-A	19	9	31	5	4	0	68
SWU13551-B	3	50	0	1	0	5	59
SWU13677-C	0	19	0	9	0	0	28
SWU13895-B	14	29	0	0	0	0	43
SWU14461-A	20	24	12	1	4	0	61
SWU14590-A	2	56	32	10	0	0	100
SWU14590-C	2	54	32	10	0	0	98
SWU14654-C	10	25	0	5	4	0	44
SWU14702-A	10	30	32	10	4	0	86
SWU14818-B	2	1	1	1	0	0	5
SWU14961-C	4	18	0	10	4	0	36
SWU15000-A	12	49	32	10	4	0	107
SWU15194-G	7	18	0	0	4	1	30
SWU15212-B	20	54	32	1	0	0	107
SWU15642-E	5	13	0	0	4	0	22
SWU16373-D	12	30	0	0	4	0	46
SWU16441-C	0	5	15	0	0	0	20
SWU16671-D	4	2	0	0	0	0	6
SWU16802-B	6	9	0	1	0	0	16
SWU18252-B	20	56	31	10	0	0	117
SWU18262-E	1	11	2	3	0	0	17
SWU18289-B	7	2	32	10	4	0	55
Common Loci	**88**	**101**	**82**	**91**	**67**	**30**	
