# Supplementary material for: Assessment of Genetic Diversity, Population Structure, and Evolutionary Relationship of Uncharacterized Genes in a Novel Germplasm Collection of Diploid and Allotetraploid Gossypium Accessions Using EST and Genomic SSR Markers
Source: Int J Mol Sci. 2018 Aug 14;19(8):2401. doi: 10.3390/ijms19082401 (PMC6121227; doi:10.3390/ijms19082401)
Supplement: Supplementary file 1 [file ijms-19-02401-s001.zip › ijms-326291-suppl-final/Supplementary Table S5.pdf]

Supplementary Table S4: Proportional Membership of cotton accessions to clusters as determined by model based analysis using STRUCTURE. Lines were assigned to a group based on membership probability higher than 0.70. The identified cluster roughly correspond to following geographical areas of cotton belt: Cluster 1-Santa Cruz (D3), cluster 2-Hawaiian, cluster 3-Isabella, cluster 4-Dominican Republic, cluster 5-San Cristobal and cluster 6-Santa Cruz (*G. barbadense*).

Sr. No.	Accession Name	Cluster 1	Cluster 2	Cluster 3	Cluster 4	Cluster 5	Cluster 6	Group
1	3-79-2	0.000	0.000	0.000	0.000	0.000	0.998	GP6
2	3-79-3	0.000	0.000	0.001	0.000	0.002	0.996	GP6
3	AD2-1-1 GLPS	0.000	0.000	0.000	0.000	0.000	0.999	GP6
4	AD2-1-2 GLPS	0.000	0.000	0.000	0.000	0.000	0.999	GP6
5	AD2-1-3 GLPS	0.000	0.000	0.000	0.000	0.000	0.999	GP6
6	AD2-1-4 GLPS	0.000	0.000	0.000	0.000	0.000	0.999	GP6
7	AD2-1-5 GLPS	0.001	0.000	0.000	0.001	0.000	0.998	GP6
8	AD2-6	0.001	0.031	0.001	0.001	0.337	0.629	GP6
9	AD2-2 GLPS	0.000	0.000	0.001	0.001	0.034	0.964	GP6
10	AD2-3-1 GLPS	0.000	0.000	0.001	0.000	0.003	0.996	GP6
11	AD2-3-2 GLPS	0.000	0.000	0.000	0.000	0.000	0.999	GP6
12	AD2-4-1 GLPS	0.000	0.000	0.000	0.000	0.000	0.999	GP6
13	AD2-4-2 GLPS	0.000	0.000	0.000	0.000	0.000	0.999	GP6
14	AD2-5-1 GLPS	0.000	0.000	0.002	0.000	0.001	0.996	GP6
15	AD2-5-2GLPS	0.000	0.000	0.000	0.000	0.000	0.998	GP6
16	AD2-5-3 GLPS	0.001	0.000	0.001	0.001	0.002	0.996	GP6
17	AD2-5-4 GLPS	0.001	0.000	0.186	0.000	0.002	0.811	GP6
18	XH-18	0.001	0.110	0.104	0.001	0.229	0.554	Mixed
19	XH-21	0.011	0.142	0.135	0.002	0.242	0.468	Mixed
20	Sibruk Egyptian	0.001	0.121	0.002	0.001	0.269	0.606	Mixed
21	AD5-lz	0.009	0.000	0.674	0.071	0.001	0.243	Mixed
22	AD5-3	0.097	0.004	0.805	0.014	0.010	0.070	GP3
23	AD5-7	0.035	0.000	0.624	0.008	0.121	0.211	Mixed
24	AD5-25-1	0.000	0.001	0.492	0.000	0.418	0.088	Mixed
25	AD5-26-1	0.001	0.001	0.629	0.000	0.120	0.247	Mixed
26	AD5-26-2	0.002	0.001	0.647	0.000	0.113	0.236	Mixed
27	AD5-28-1	0.000	0.000	0.998	0.000	0.001	0.000	GP3
28	AD5-28-2	0.000	0.000	0.999	0.000	0.000	0.000	GP3
29	AD5-28-3	0.000	0.000	0.998	0.000	0.000	0.000	GP3
30	AD5-28-4	0.000	0.000	0.999	0.000	0.000	0.000	GP3
31	AD5-29-1	0.001	0.000	0.996	0.000	0.001	0.002	GP3
32	AD5-29-2	0.000	0.001	0.983	0.001	0.015	0.000	GP3
33	AD5-30-1	0.000	0.000	0.999	0.000	0.000	0.000	GP3
34	AD5-30-2	0.000	0.000	0.999	0.000	0.000	0.000	GP3
35	AD5-30-3	0.000	0.000	0.999	0.000	0.000	0.000	GP3

36	AD5-30-4	0.001	0.000	0.997	0.000	0.001	0.000	GP3
37	AD5-31-1	0.002	0.001	0.994	0.000	0.002	0.001	GP3
38	AD5-31-2	0.001	0.000	0.997	0.001	0.001	0.001	GP3
39	AD5-31-3	0.001	0.000	0.947	0.001	0.049	0.002	GP3
40	AD5-43	0.036	0.001	0.515	0.005	0.443	0.001	Mixed
41	AD5-48	0.000	0.000	0.611	0.020	0.367	0.002	Mixed
42	AD5-50	0.001	0.001	0.634	0.001	0.362	0.000	Mixed
43	AD5-51	0.003	0.001	0.578	0.001	0.416	0.001	Mixed
44	AD5-52	0.000	0.000	0.570	0.000	0.429	0.000	Mixed
45	AD5-53	0.000	0.000	0.490	0.000	0.508	0.001	Mixed
46	AD5-54-1	0.053	0.073	0.001	0.002	0.843	0.028	GP5
47	AD5-54-2	0.001	0.001	0.007	0.049	0.937	0.006	GP5
48	AD5-55	0.002	0.000	0.002	0.001	0.827	0.168	GP5
49	AD5-22	0.002	0.000	0.532	0.001	0.328	0.137	Mixed
50	AD5-24	0.017	0.001	0.556	0.010	0.383	0.033	Mixed
51	AD5-25-2	0.000	0.001	0.487	0.000	0.456	0.055	Mixed
52	AD5-32	0.002	0.002	0.373	0.001	0.265	0.356	Mixed
53	AD5-32-1	0.002	0.002	0.374	0.001	0.267	0.353	Mixed
54	AD5-33	0.001	0.001	0.415	0.000	0.464	0.119	Mixed
55	AD5-34-1	0.000	0.000	0.681	0.000	0.317	0.002	Mixed
56	AD5-34-2	0.001	0.001	0.695	0.000	0.291	0.012	Mixed
57	AD5-34-3	0.000	0.002	0.669	0.001	0.228	0.100	Mixed
58	AD5-34-4	0.000	0.001	0.626	0.000	0.240	0.132	Mixed
59	AD5-34-5	0.003	0.014	0.415	0.001	0.477	0.089	Mixed
60	AD5-35-1	0.001	0.001	0.463	0.001	0.315	0.220	Mixed
61	AD5-35-2	0.004	0.001	0.469	0.001	0.306	0.220	Mixed
62	AD5-56	0.000	0.015	0.002	0.040	0.940	0.003	GP5
63	AD5-57	0.104	0.003	0.006	0.000	0.885	0.001	GP5
64	AD5-58	0.001	0.000	0.065	0.003	0.931	0.001	GP5
65	AD5-59-1	0.000	0.000	0.000	0.000	0.999	0.000	GP5
66	AD5-59-2	0.001	0.001	0.001	0.000	0.998	0.000	GP5
67	AD5-60	0.000	0.000	0.000	0.000	0.999	0.000	GP5
68	AD5-61-1	0.000	0.000	0.000	0.000	0.998	0.001	GP5
69	AD5-61-2	0.000	0.000	0.001	0.000	0.998	0.000	GP5
70	AD5-61-3	0.000	0.000	0.000	0.000	0.999	0.000	GP5
71	AD5-61-4	0.000	0.000	0.000	0.000	0.999	0.000	GP5
72	AD5-61-5	0.000	0.000	0.000	0.000	0.999	0.000	GP5
73	AD5-61-6	0.026	0.000	0.004	0.000	0.968	0.001	GP5
74	AD5-61-7	0.000	0.009	0.001	0.004	0.982	0.004	GP5
75	AD5-61-8	0.000	0.000	0.000	0.000	0.999	0.000	GP5
76	AD5-61-9	0.016	0.000	0.000	0.001	0.983	0.000	GP5
77	AD5-61-10	0.000	0.002	0.001	0.000	0.995	0.002	GP5
78	Subsp.darwini II-1	0.001	0.002	0.655	0.002	0.006	0.334	Mixed
79	Subsp.darwini II-2	0.001	0.019	0.596	0.001	0.096	0.288	Mixed
80	D3-k-lz	0.998	0.001	0.001	0.000	0.000	0.000	GP1
81	D3k-21-1	0.999	0.000	0.000	0.000	0.000	0.000	GP1
82	D3k-21-2	0.999	0.000	0.000	0.000	0.000	0.000	GP1
83	D3k-21-3	0.999	0.000	0.000	0.000	0.000	0.000	GP1

84	D3k-21-4	0.999	0.000	0.000	0.000	0.000	0.000	GP1
85	CCRI12	0.111	0.278	0.010	0.472	0.011	0.117	Mixed
86	CCRI16	0.122	0.245	0.001	0.507	0.026	0.097	Mixed
87	AD3-27	0.002	0.874	0.001	0.025	0.098	0.000	GP2
88	AD3-28-1	0.004	0.989	0.000	0.001	0.006	0.000	GP2
89	AD3-28-2	0.000	0.998	0.000	0.000	0.001	0.000	GP2
90	AD3-30-1	0.000	0.999	0.000	0.000	0.000	0.000	GP2
91	AD3-30-2	0.000	0.999	0.000	0.000	0.000	0.000	GP2
92	AD3-lz	0.000	0.999	0.000	0.000	0.000	0.000	GP2
93	AD3-70	0.001	0.998	0.000	0.000	0.000	0.000	GP2
94	AD3-71	0.000	0.999	0.000	0.000	0.000	0.000	GP2
95	AD3-72	0.000	0.999	0.000	0.000	0.000	0.000	GP2
96	AD3-73	0.000	0.999	0.000	0.000	0.000	0.000	GP2
97	AD3-68	0.001	0.997	0.001	0.000	0.001	0.000	GP2
98	AD3-69	0.000	0.995	0.002	0.001	0.001	0.001	GP2
99	AD3-21	0.000	0.999	0.000	0.000	0.000	0.000	GP2
100	AD3-23	0.000	0.999	0.000	0.000	0.000	0.000	GP2
101	AD3-24	0.000	0.999	0.000	0.000	0.000	0.000	GP2
102	AD3-25	0.000	0.999	0.000	0.000	0.000	0.000	GP2
103	AD3-26	0.000	0.999	0.000	0.000	0.000	0.000	GP2
104	AD3-32	0.000	0.999	0.000	0.000	0.000	0.000	GP2
105	AD3-33	0.000	0.999	0.000	0.000	0.000	0.000	GP2
106	AD3-35	0.000	0.999	0.000	0.000	0.000	0.000	GP2
107	AD3-36	0.000	0.999	0.000	0.000	0.000	0.000	GP2
108	AD3-37	0.017	0.981	0.001	0.000	0.001	0.000	GP2
109	AD3-38	0.000	0.998	0.000	0.000	0.000	0.000	GP2
110	AD3-39	0.000	0.999	0.000	0.000	0.000	0.000	GP2
111	AD3-40	0.010	0.987	0.000	0.001	0.001	0.000	GP2
112	AD3-41	0.000	0.999	0.000	0.000	0.000	0.000	GP2
113	AD3-42	0.000	0.998	0.000	0.001	0.000	0.001	GP2
114	AD3-45	0.000	0.999	0.000	0.000	0.000	0.000	GP2
115	AD3-46	0.000	0.999	0.000	0.000	0.000	0.000	GP2
116	AD3-47	0.001	0.998	0.000	0.000	0.000	0.000	GP2
117	AD3-49	0.001	0.996	0.000	0.002	0.000	0.000	GP2
118	AD3-50	0.006	0.964	0.005	0.023	0.001	0.000	GP2
119	AD6-01-1	0.002	0.000	0.000	0.997	0.000	0.000	GP4
120	AD6-01-2	0.000	0.000	0.000	0.999	0.000	0.000	GP4
121	AD6-01-3	0.000	0.000	0.000	0.999	0.000	0.000	GP4
122	AD6-02-4	0.002	0.002	0.000	0.994	0.001	0.001	GP4
123	AD6-03-7	0.000	0.000	0.000	0.999	0.000	0.000	GP4
124	AD6-03-8	0.000	0.000	0.000	0.999	0.000	0.000	GP4
125	AD6-03-9	0.000	0.000	0.000	0.999	0.000	0.000	GP4
126	AD6-03-10	0.000	0.001	0.000	0.998	0.000	0.000	GP4
127	AD6-03-11	0.000	0.000	0.000	0.999	0.000	0.000	GP4
128	AD6-03-12	0.000	0.061	0.001	0.871	0.066	0.000	GP4
129	AD7-01-13	0.001	0.344	0.002	0.651	0.001	0.001	Mixed
130	AD7-01-14	0.002	0.337	0.002	0.656	0.002	0.001	Mixed
131	AD7-01-15	0.002	0.321	0.002	0.673	0.002	0.001	Mixed

132	AD7-01-16	0.001	0.336	0.002	0.658	0.002	0.001	Mixed
