# Supplementary figures and images for: Assessment of Genetic Diversity, Population Structure, and Evolutionary Relationship of Uncharacterized Genes in a Novel Germplasm Collection of Diploid and Allotetraploid Gossypium Accessions Using EST and Genomic SSR Markers
Source: Int J Mol Sci. 2018 Aug 14;19(8):2401. doi: 10.3390/ijms19082401 (PMC6121227; doi:10.3390/ijms19082401)

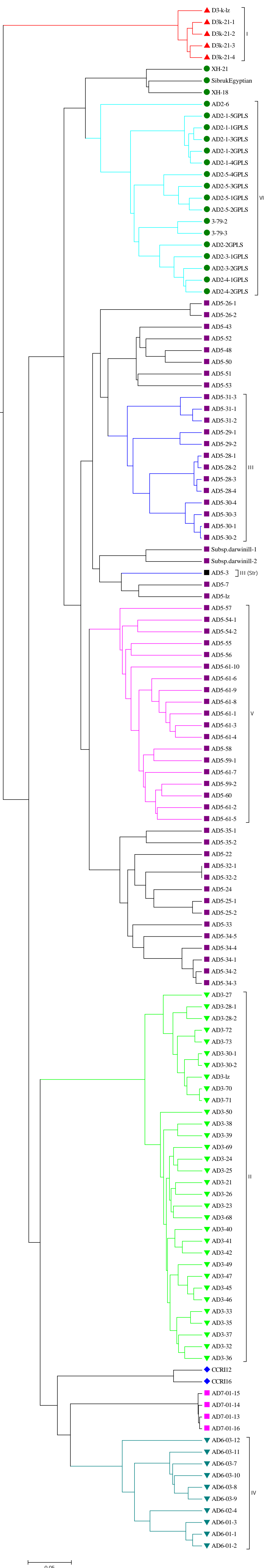

Supplement: Supplementary file 1 [file ijms-19-02401-s001.zip › ijms-326291-suppl-final/Supplementary fig 1.pdf]
